# Rational Design of High-Performance PEO/Ceramic Composite Solid Electrolytes for Lithium Metal Batteries

**DOI:** 10.1007/s40820-023-01055-z

**Published:** 2023-03-31

**Authors:** Yanxia Su, Fei Xu, Xinren Zhang, Yuqian Qiu, Hongqiang Wang

**Affiliations:** State Key Laboratory of Solidification Processing, Centre for Nano Energy Materials, School of Materials Science and Engineering, Northwestern Polytechnical University, Shaanxi Joint Laboratory of Graphene (NPU), Xi’an, 710072 People’s Republic of China

**Keywords:** Composite solid electrolytes, Ionic conductivity, Interfacial compatibility, Ion conduction pathways, Li metal batteries

## Abstract

Composite solid electrolytes (CSEs) with poly(ethylene oxide) (PEO) have become fairly prevalent for fabricating high-performance solid-state lithium metal batteries due to their high Li^+^ solvating capability, flexible processability and low cost. However, unsatisfactory room-temperature ionic conductivity, weak interfacial compatibility and uncontrollable Li dendrite growth seriously hinder their progress. Enormous efforts have been devoted to combining PEO with ceramics either as fillers or major matrix with the rational design of two-phase architecture, spatial distribution and content, which is anticipated to hold the key to increasing ionic conductivity and resolving interfacial compatibility within CSEs and between CSEs/electrodes. Unfortunately, a comprehensive review exclusively discussing the design, preparation and application of PEO/ceramic-based CSEs is largely lacking, in spite of tremendous reviews dealing with a broad spectrum of polymers and ceramics. Consequently, this review targets recent advances in PEO/ceramic-based CSEs, starting with a brief introduction, followed by their ionic conduction mechanism, preparation methods, and then an emphasis on resolving ionic conductivity and interfacial compatibility. Afterward, their applications in solid-state lithium metal batteries with transition metal oxides and sulfur cathodes are summarized. Finally, a summary and outlook on existing challenges and future research directions are proposed.

## Highlights

- The design, preparation and application of poly(ethylene oxide) (PEO)/ceramic composite solid electrolytes (CSEs) are summarized from “ceramic in polymer” and “polymer in ceramic”.
- The summary and outlook on existing challenges and future research directions of PEO/ceramic CSEs for lithium metal batteries are proposed.

## Graphical abstract

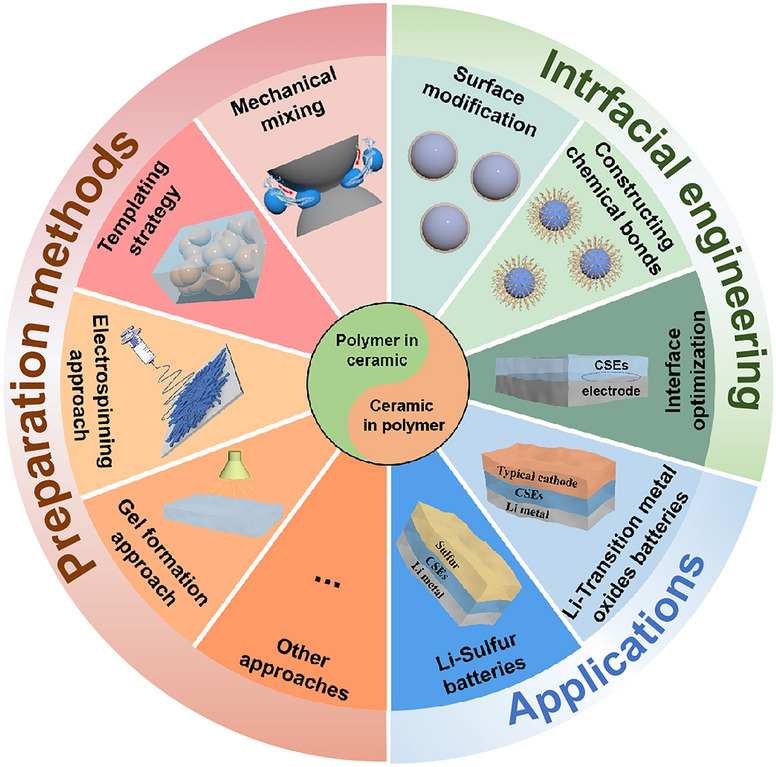

## Introduction

Currently, clean and sustainable energy is one of the most important issues for economic development worldwide [1]. Thus, energy storage and conversion are becoming more indispensable than ever before, especially with the fast increase in global population and worldwide development [2–5]. Rechargeable lithium-ion batteries (LIBs) have a high gravimetric and volumetric energy density compared with the other types of commercially available rechargeable battery technologies for electrochemical energy storage, and thus they have been extensively used in portable devices, electric vehicles, and grid energy storage since their first commercialization by Sony in 1991 [6–8]. However, state-of-the-art commercial LIB devices gradually cannot meet the increasing demand for powering a range of electric vehicles and large-scale grid energy storage [9, 10], due to the limited energy density (˂ 200 Wh kg^−1^) with current electrode materials like LiFePO_4_ cathode and graphite anode. Compared to the theoretical capacity of 372 mAh g^−1^ for the graphite anode in commercial LIBs, the lithium metal anode shows a much higher theoretical capacity (3860 mAh g^−1^). Meanwhile, it shows the lowest reduction potential (− 3.04 V vs. standard hydrogen electrode), thus allowing a high voltage in full battery devices [11, 12]. Consequently, lithium metal batteries (LMBs) with Li metal anodes could achieve a much higher energy density originating from both large capacity and high full cell voltage windows. However, the practical use of LMBs is seriously hindered by uncontrollable dendrite growth, unstable electrolyte/Li interface and thus poor cycle stability [10, 13]. Meanwhile, the flammable and volatile organic liquid electrolytes used in conventional LMBs give rise to safety concerns (e.g., fire or explosion), especially with the occurrence of short circuits induced by Li dendrite formation [14–16]. Furthermore, they also show unsatisfactory stability against high voltage cathodes (> 4.5 V) and suffer from side reactions, aggravating the capacity and lifespan degradation [17, 18]. Therefore, it is necessary to develop efficient electrolyte systems beyond the liquid ones, which could allow dendrite-free Li anodes and safe operation while delivering long-cycle stability and tolerance to high voltage cathodes.

In this context, solid-state electrolytes and gel electrolytes have recently drawn sufficient attention as a potential substitute for organic liquid electrolytes in terms of safety and Li dendrite suppression [19–21]. Gel electrolytes have higher ionic conductivity due to the presence of liquid component, but poor mechanical strength and the possibility of uncontrolled thermal runaway. However, solid-state electrolytes have sufficient mechanical strength and higher security [22, 23]. Therefore, solid-state electrolytes could revive the possibility of using a high-energy-density Li metal anode, which is mainly divided into three categories according to their composition: solid ceramic electrolytes [24, 25], solid polymer electrolytes [26, 27] and composite solid electrolytes (CSEs) [28–30]. CSEs are unique in that they can merge the advantages of both solid ceramic electrolytes and solid polymer electrolytes, thus exhibiting better ionic conductivity, higher compatibility with electrodes and enhanced mechanical tolerance [31, 32]. A typical CSE is composed of polymers solvating lithium salts and inorganic ceramics in various architectures [33]. Inorganic ceramics can be either Li^+^ insulative, such as TiO_2_ [34, 35], Al_2_O_3_ [36], SiO_2_ [37] or Li^+^ conductive, including Li_1.3_Al_0.3_Ti_1.7_(PO_4_)_3_ (LATP) [38, 39], Li_10_GeP_2_S_12_(LGPS) [40], Li_7_La_3_Zr_2_O_12_(LLZO) [41, 42], LiTa_2_PO_8_ (LTPO) [43], etc. Various ceramic matrix/fillers exhibit different effects on CSEs due to the nature of ceramics. Form the viewpoint of ionic conductivity, conductive ceramics work better than insulative ones. However, Li^+^ insulative ceramic fillers generally show the advantages of low cost and adaptable processability. For those conductive ceramics sulfides exhibit ionic conductivity up to 10^–3^ S cm^−1^ and low grain boundary resistance at room temperature due to the large size and polarization of sulfide ions. In contrast, oxides (LLZO, LIPON) have better oxidation resistance than sulfides due to kinetic stability, and garnet LLZO (cubic phase) shows the highest resistance to being reduced by lithium. The polymers used generally include poly(vinylidene fluoride) (PVDF) [44, 45], poly(ethylene oxide) (PEO) [46, 47], polyacrylonitrile (PAN) [37], poly(vinylidene fluoride-hexafluoropropylene) (PVDF–HFP), etc. [48], and Li salts include LiClO_4_ [49], LiTFSI [50], LiFSI [51], etc. PVDF has good dielectric constant and high interfacial stability with lithium metal, while possess lower ionic conductivity at room temperature [52]. PAN has good stability with high-voltage cathode, but poor stability with lithium metal [53]. Among various CSEs, polymers using PEO are unique in that they possess higher Li^+^ solvating capability, flexibility, processability and low cost [27].

Since the first discovery of the ionic conductivity of PEO by Wright et al. [54] in 1973, PEO-based solid-state electrolytes have been extensively studied [55, 56]. The transportation of Li^+^ is mainly dominated by the segmental relaxation of PEO chains, and thus, the high crystallinity of PEO gives rise to the limited ionic conductivities (< 10^–5^ S cm^−1^) and low Li^+^ transference numbers (0.2–0.4) at room temperature [57]. Meanwhile, the poor mechanical strength and the vulnerability against oxidation of PEO (no more than 4.0 V) further impede their practical applications, especially for high-voltage solid-state LMBs [58, 59]. To overcome these shortcomings, the combination of ceramics and PEO is anticipated to achieve better electrolytes delivering simultaneously attractive ionic conductivity, high voltage tolerance and good mechanical strength. As early as 1998, Croce et al. [60] firstly demonstrated that the Li^+^ conductivity can be improved to 10^–4^ S cm^−1^ at 50 °C and 10^–5^ S cm^−1^ at 30 °C by incorporating TiO_2_ and Al_2_O_3_ as filler into PEO with LiClO_4_ salt, respectively. Encouraged by this pioneering work, much effort has been devoted to optimizing the ionic conductivity as well as the compatibility for practical CSEs in LMBs [61, 62].

**Fig. 1:**
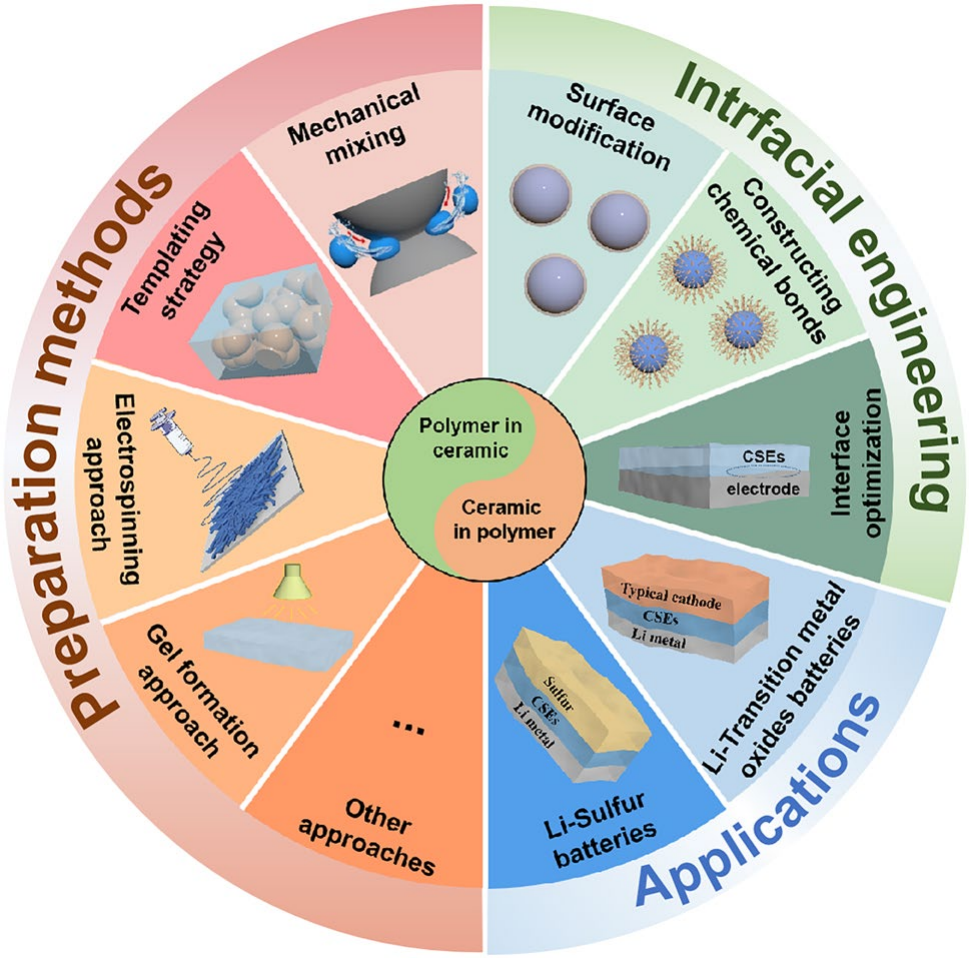
The organization of this review scheme

Benefitting from these advantages of PEO, such as, low density and interface impedance, easy to thin layer and machining, PEO and its derived CSEs become one of the most commercial prospect electrolyte materials. The PEO electrolyte was the first to promote commercialization by Bollore [63] Company in 2011, achieving the electric vehicle with solid-state battery as the power system (110 Wh kg^−1^ at 70–80 °C). Unfortunately, low ionic conductivity at room temperature limits energy density and enables energy consumption and cost after heating up. Therefore, a review focused on discussing the design, preparation and application of PEO/ceramic CSEs is required, especially for identifying conduction mechanisms and realizing performance optimization, furtherly promoting cost-efficient production. Thus, we specifically reviewed PEO/ceramic CSEs, which is different from the recent large number of reviews focusing on various polymer/ceramic CSEs [29, 57, 62, 64–70]. In this review, beginning with a brief introduction to solid-state electrolytes and especially for PEO/ceramic CSEs, the Li^+^ conduction mechanism is analyzed with architectures from “ceramic in polymer” to “polymer in ceramic”. Then, preparation methods are summarized, including mechanical mixing, templating strategies, electrospinning and gel formation approaches, along with discussions of their respective cons and pros for developing high-quality PEO/ceramic CSEs. Subsequently, interface regulation and architecture design within PEO/ceramic CSEs and between CSEs/electrodes are emphasized to optimize interfacial compatibility (Fig. 1). After that, the applications of PEO/ceramic CSEs paired with transition metal oxides and sulfur cathodes in solid-state lithium metal batteries are provided. Finally, a summary and outlook of PEO/ceramic CSEs are proposed. Hopefully, this review will engender interest in acquiring a basic understanding of PEO-based CSEs and stimulate further explorations even for beginners in the ion conduction mechanism, design strategies and Li metal full cells with high ion conductivity, superior compatible with cathode and anode, and ultrathin thickness yet mechanical stability.

## Li^+^ Conduction Mechanism

The Li^+^ conductivity is one of the most important parameters in CSEs. An insightful understanding of the Li^+^ conduction mechanism or behavior is critical to realizing high ionic conductivity, which is closely associated with the relative ratio and architecture of PEO and ceramic [71–73]. Moreover, advanced characterization techniques are key to studying the local structural environments and dynamics of lithium ions.

### Effects of Architecture on Li^+^-Conducting Pathways

The conduction mechanism of PEO–Li can be generalized by the combination and dissociation of EO–Li bonds, together with the main chain movement of PEO. Li^+^ can be transported on a single chain or between different chains. The Li^+^ migration depends on the movement of polymer chains and mainly occurs in the amorphous region. However, PEO is easy to form crystalline phase at room temperature, which hinders chain movement and thus Li^+^ migration. Therefore, the operating temperature of solid-state batteries is usually higher than the melting temperature of PEO (˃ 65 °C) [66]. However, PEO homopolymer is a viscous liquid, and its mechanical properties are too weak to slow down the growth of lithium dendrites during cycling. Many methods have been explored to improve ionic conductivity and mechanical properties of PEO-based solid polymer electrolytes. It is found that the adding of inorganic fillers can promote the formation of local amorphous regions, thus promoting Li^+^ transfer [74].

**Fig. 2:**
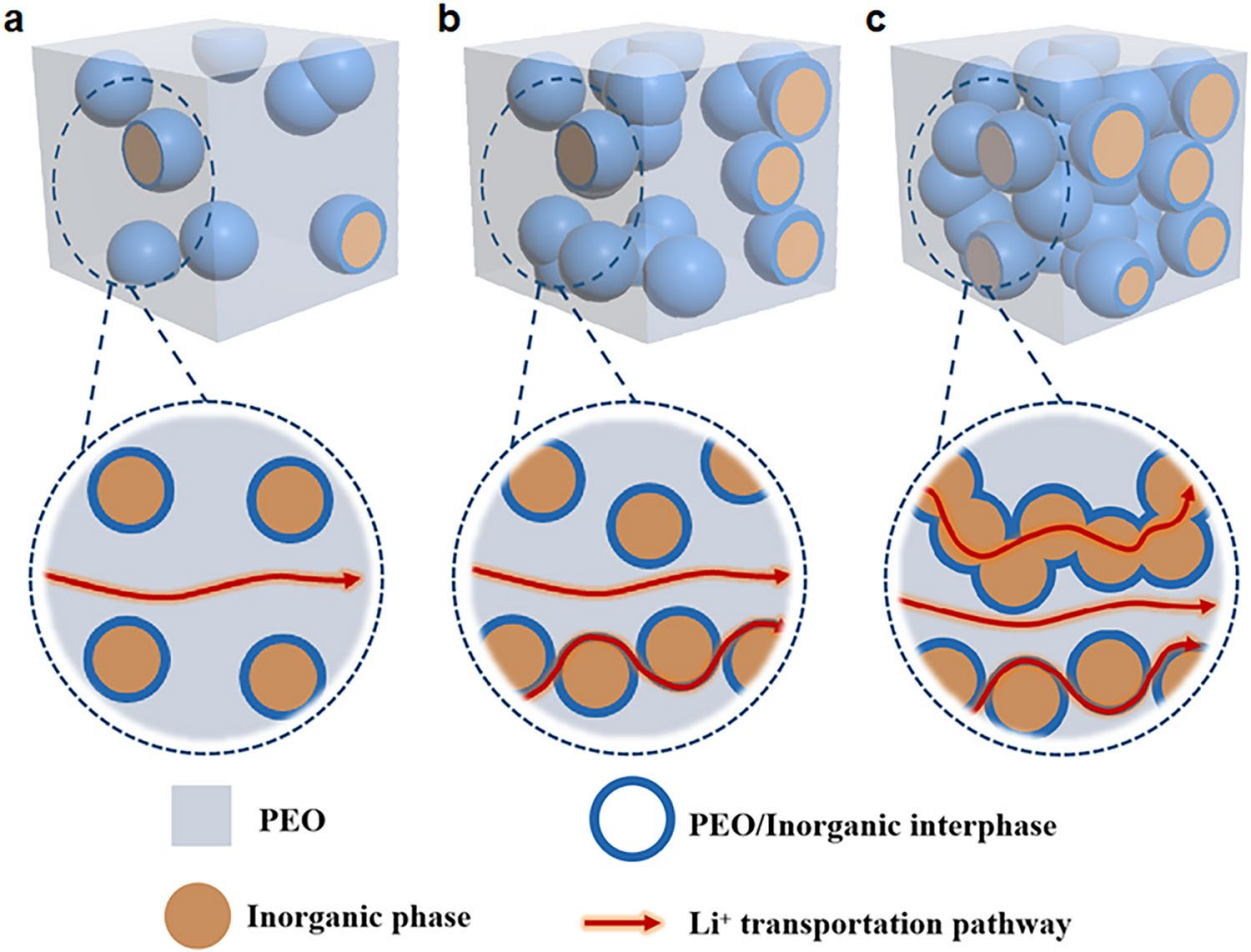
Schematic illustration of the Li^+^ conduction pathways **a** in the PEO phase, **b** in the PEO phase and at PEO/ceramic interface, and **c** in the PEO phase and ceramic phase and at PEO/ceramic interface

According to the report of Goodenough, CSEs can be divided into two categories, “ceramic in polymer” and “polymer in ceramic” [75]. In the former system, ceramic serves as a filler (minor phase) in the PEO–Li salt complex matrix to increase the Li^+^ conductivity, whereas in the latter system, the addition of PEO–Li salt in the ceramic matrix can improve interface compatibility [38, 76]. The possible Li^+^ conduction pathways and conduction properties are summarized in Fig. 2, as discussed below [77, 78]. Generally, there are three possible Li^+^ conduction pathways, including in the PEO phase, at PEO/ceramic interface and in the ceramic phase [79].

In the “ceramic in polymer” system, ceramic fillers help to suppress the crystallization of PEO chains [77, 80]. The lower the crystallinity is, the higher the Li^+^ transportation ability because better crystallinity could decrease the free volume with more compact packing of parallel polymer chains against Li^+^ transportation [33, 61]. Therefore, the incorporation of fillers in PEO could enhance the ionic conductivity by the generation of more amorphous regions for facile segment movement. Fillers such as nanoparticles are generally discontinuously dispersed in the PEO matrix (Fig. 2a). In this context, Li^+^ conduction pathways are mainly governed by the motion within the PEO phase, no matter whether the fillers are inert or conductive for lithium ions [28, 57]. This was confirmed experimentally by increasing the fraction of ceramics in CSEs, in which Li^+^ conduction pathways transfer from PEO to the PEO/ceramic interface, which plays a vital role in regulating Li^+^ conduction in PEO-based CSEs (Fig. 2b) [77]. In addition to the typical nanoparticle fillers, different morphologies, such as nanowire and nanosheet fillers, have also been used [81]. It was reported that nanowire fillers not only favor the generation of amorphous regions but also offer continuous active pathways along the interfaces for fast Li^+^ transportation and superior ionic conductivity compared to nanoparticles [82, 83]. Moreover, well-aligned inorganic Li^+^ conductive nanowires were reported to show favorable for Li^+^ conduction than randomly aligned nanowires [84]. Nanosheet fillers such as C_3_N_4_ [85], BN [86], MXene [81, 87] or vermiculite [88] have been used, which are expected to offer a higher surface area and provide continuous 2D interfaces between the fillers and the polymer matrix, thus ensuring rapid diffusion of lithium ions [89–91]. Exploration of a broader range of nanosheet fillers is still needed with both electron insulation and chemical and thermal stability. The Li^+^ conduction pathways in the “ceramic in polymer” configuration will influence the Li^+^ conductivity. Generally, with increasing ceramic content, the ionic conductivity increases. The ionic conductivity reaches the maximum at the percolation threshold, such as 10 wt% LLZTO [75], 25 wt% LATP [92], 20 wt% LAGP [93], 5 wt% MnO_2_ [90] and 7.5 wt% TiO_2_ [94]. However, further increasing the ceramic content will block ion transmission and cause a decrease in ionic conductivity. Bouchet et al. [95] also verified the conclusion via using CSEs model system. The size of ceramic fillers also influences the percolation threshold [96]. Compared with corresponding bulk fillers, nanofillers could provide a higher surface area, sufficient interfacial contact sites with PEO, and thus a low percolation effect and smooth ion conduction pathways. For example, Liu et al. [97] developed 200 nm LLZTO and found that the percolation threshold is 20 wt% showing the best ionic conductivity. Likewise, a 100 nm LLZTO nanoparticle as filler shows a value of 11.53 wt% [98]. Recently, we prepared 8.3 nm LLZTO nanoparticles by laser manufacturing in liquid, and the percolation threshold is down to 2 wt%, which is one of the record values among PEO-based electrolytes, to the best of our knowledge [80]. Apart from the above ceramic content and size, Li-salt content and ceramic species also impact the Li^+^ conduction mechanisms. For example, the impact of LiTFSI content (EO/Li = 18:1, 9:1 and 6:1) on LGPS/PEO interface formation showed that low LiTFSI content has little effect on interface formation. The high LiTFSI content also has limited LGPS/PEO interface formation. This is because excess LiTFSI partially aggregates and the interaction between PEO and LiTFSI alters mechanical properties of PEO, showing poor interface compatibility with LGPS [72]. As compared with the Li^+^ insulating ceramics, Li^+^-conducting ones improve Li^+^ conductivity due to their ability to conduct Li^+^ through the ceramic as well as across the interface [99]. Meanwhile, the Li^+^ conductive filler LiZr_2_(PO_4_)_3_ (LZP) allows more Li^+^ reallocation in the disordered local environment of PEO, facilitating better Li^+^ mobility and enhancing the ionic conductivity, superior to Li^+^ insulative Al_2_O_3_ filler CSEs [78].

In contrast, in the “polymer in ceramic” system, PEO–Li salt complexes are embedded or confined into the continuous, tightly packed ceramic matrix (Fig. 2c). In most cases, ceramic matrix is Li^+^ conductive, which not only helps suppress the crystallization of the PEO phase but also affords continuous Li^+^ conduction pathways within the ceramic matrix and along the interfaces [43, 80]. Only in some few cases, the ceramics (e.g., SiO_2_) is Li^+^-insulative, and thus the Li^+^ is mainly transported along the continuous interface [100]. In these “polymer in ceramic” architectures, the continuous 3D garnet skeleton avoids agglomeration of ceramic particles and provides continuous conductive interfaces, thus improving the conductivity as compared with the isolated particles in polymer matrix, at their respective percolation threshold. For example, Guo et al. [101] reported that CSEs with 3D garnet skeleton showed an ionic conductivity of 1.2 × 10^–4^ S cm^−1^ at 30 °C, which was about two times that of garnet particle-reinforced polymer-based CSEs (6.4 × 10^–5^ S cm^−1^).

### Characterization Techniques

**Fig. 3:**
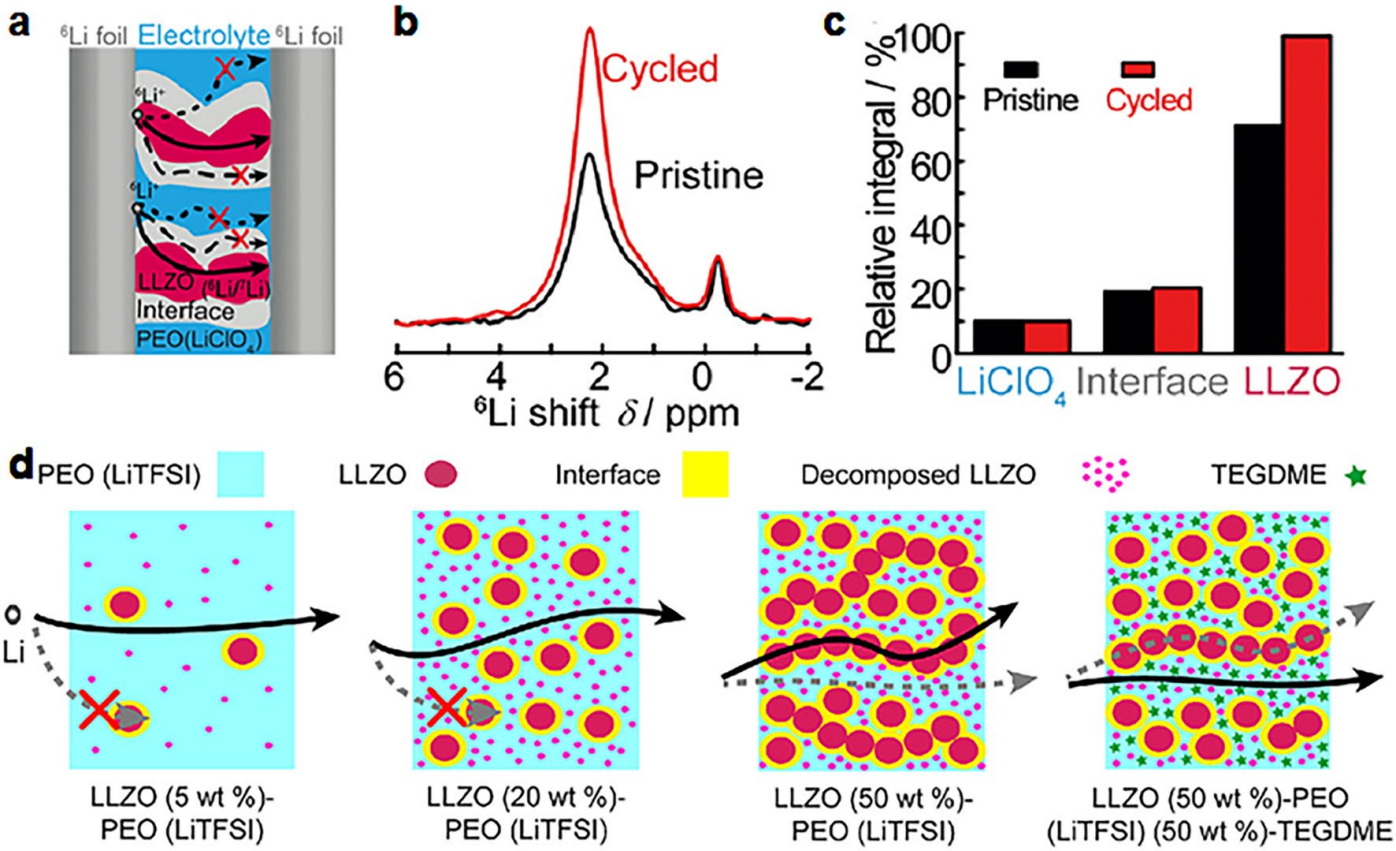
**a** Illustration of the symmetric ^6^Li battery and possible Li^+^ conduction pathways. **b** Comparison of the ^6^Li spectra of the LLZO/PEO(LiClO_4_) electrolytes before and after cycling. **c** Quantitative analysis of the ^6^Li amount in LiClO_4_, the interface, and LLZO of LLZO/PEO(LiClO_4_) before and after cycling. **a**–**c** Reproduced with permission [104]. Copyright 2016, Wiley–VCH. **d** Schematic of Li^+^ pathways within CSEs. Reproduced with permission [77]. Copyright 2018, American Chemical Society

Various characterization tools are used to clarify Li^+^ conduction pathways. Solid-state Li nuclear magnetic resonance (NMR) is one of the most powerful tools for studying local structural environments and the dynamic process of Li^+^ within CSEs using isotope exchange [102, 103]. This is realized by using a ^6^Li labeled foil electrode to trace the ^6^Li distribution in the original ^7^Li-based CSEs via Li NMR due to the partial replacement of ^7^Li with ^6^Li during each cycle, thus enabling the clarification of Li^+^ conduction pathways. As early as 2016, Hu et al. [104] reported the first experimental evidence of Li^+^ conduction pathways using selective isotope labeling of solid-state Li NMR, and the result shows that Li^+^ transportation prefers to go through the LLZO ceramic phase rather than the PEO/LLZO interface or PEO phase with 50 wt% LLZO (Fig. 3a–c). The LLZO content is critical to determine the Li^+^ conduction pathways. As shown in Fig. 3d, Li^+^ conduction pathways gradually transfer from the PEO phase to the percolated network made of loosely connected LLZO particles, by increasing the LLZO content (from 5 to 50 wt%) in CSEs [77]. Intriguingly, under 50 wt% LLZO in CSEs, the Li^+^ conduction pathways can switch from the LLZO to the PEO phase by adding tetraethylene glycol dimethyl ether as a liquid plasticizer, which helps to increase the ion mobility in the PEO phase (Fig. 3d). When the amount of the ceramic LGPS further increases to 70 wt% reaching the percolated network value, the Li^+^ conduction pathways are in both LGPS and interfaces, different from 50% of LGPS with Li-ion transport mainly in LGPS. However, further increasing the LGPS content to 90%, the Li^+^ transport mainly switches back to LGPS [105].

Furthermore, some in situ and ex situ characterization methods have been engaged to assist in the research of solid-state batteries, mainly including energy dispersive X-ray [106], scanning electron microscopy (SEM) [107], transmission electron microscopy (TEM) [108], Raman spectroscopy [109] and neutron depth profiling [103, 110]. Among those, in situ TEM is frequently employed with a high spatial resolution for studying the characteristics of the morphological evolution, phase transformations, chemical composite changes, interfacial behavior and solid electrolyte interphase and cathode electrolyte interphase formation in solid-state batteries, which has been documented in previous reviews for reference [111, 112].

## Preparation Methods of CSEs

The preparation methods are important to determine the architecture of CSEs and impact the ionic conductivity and electrochemical stability, which generally include mechanical mixing, templating strategies, electrospinning and gel formation approaches. Mechanical mixing is mainly applied for designing “ceramic in polymer” architectures, and composite films with thicknesses down to 30 µm can be obtained coupled with solution casting and hot pressing [113]. The templating strategy is propitious to the “polymers in ceramic” system, and the electrospinning approach is capable of fabricating nanowire membranes for both “ceramic in polymers” and “polymers in ceramic” systems. The gel formation approach can be used to design polymer-based gels for “ceramic in polymer” or ceramic-based gels for “polymer in ceramic” CSEs, which generally exhibit higher thickness (˃ 120 µm).

### Mechanical Mixing

Mechanical mixing is one of the most prevalent methods to prepare CSEs, owing to its convenience and low cost. In this strategy, the PEO, Li salt or their predissolved solution was mixed with ceramic fillers via ball milling, sonification or stirring to make a well-dispersed suspension, followed by slurry casting and drying [92, 114–118]. Control over the PEO concentration is very important. The lower concentration will lead to difficulty in forming a film during casting, while the higher concentration will result in enhanced viscosity, thus impeding the uniform dispersion of ceramic fillers in the resulting composite electrolyte film. Compared with stirring and ultrasonic mixing, ball milling is advantageous for preparing a high-concentration mixture due to its high ball milling energy. For those solid ones without any organic solvent, hot pressing of the premixed mixture is adaptable [52, 119–122]. Nevertheless, the process of solid–solid mixing makes it difficult to mix inorganic ceramics and polymers well for forming a uniform CSE film [75]. The thickness of composite films can be regulated by the viscosity of the slurry or the pressure and temperature during hot pressing, which is currently as low as 30 µm [113].

Despite the convenience and low cost, mechanical mixing suffers from the aggregation of ceramic fillers, especially for those with nanosized and high concentrations. In this regard, the ionic conductivity of CSEs cannot be efficiently enhanced due to the lack of sufficient interfaces for decreasing the PEO crystallinity. Meanwhile, filler aggregation also causes local differences in conductivity, leading to inhomogeneous Li-ion reflux in the bulk PEO substrate and thus the generation of Li dendrites [35, 123]. With the further increase in ceramic filler content (e.g., > 30 wt%), no continuous ceramic phase is generated, in contrast to the polymer in the ceramic architecture showing continuous Li^+^ conduction channels. Consequently, Li^+^ transport is blocked, causing a decrease in Li^+^ conductivity in this case [120, 124]. Therefore, there exists a percolation threshold for the ceramic filler content to realize a maximum Li^+^ conductivity, such as 2 wt% for Nano-LLZTO [80], 20 wt% for sheet-like Li_6.25_La_3_Zr_2_Al_0.25_O_12_ (LLZAO) [89], 5 wt% for SiO_2_ [125] and 25 wt% for LATP [92] and LiZr_2_(PO_4_)_3_ [78]. To pursue facilitated conductivity under heavy ceramic content, the design of polymer in ceramic architecture is necessary, as discussed below by templating strategy and others.

### Templating Strategy

**Fig. 4:**
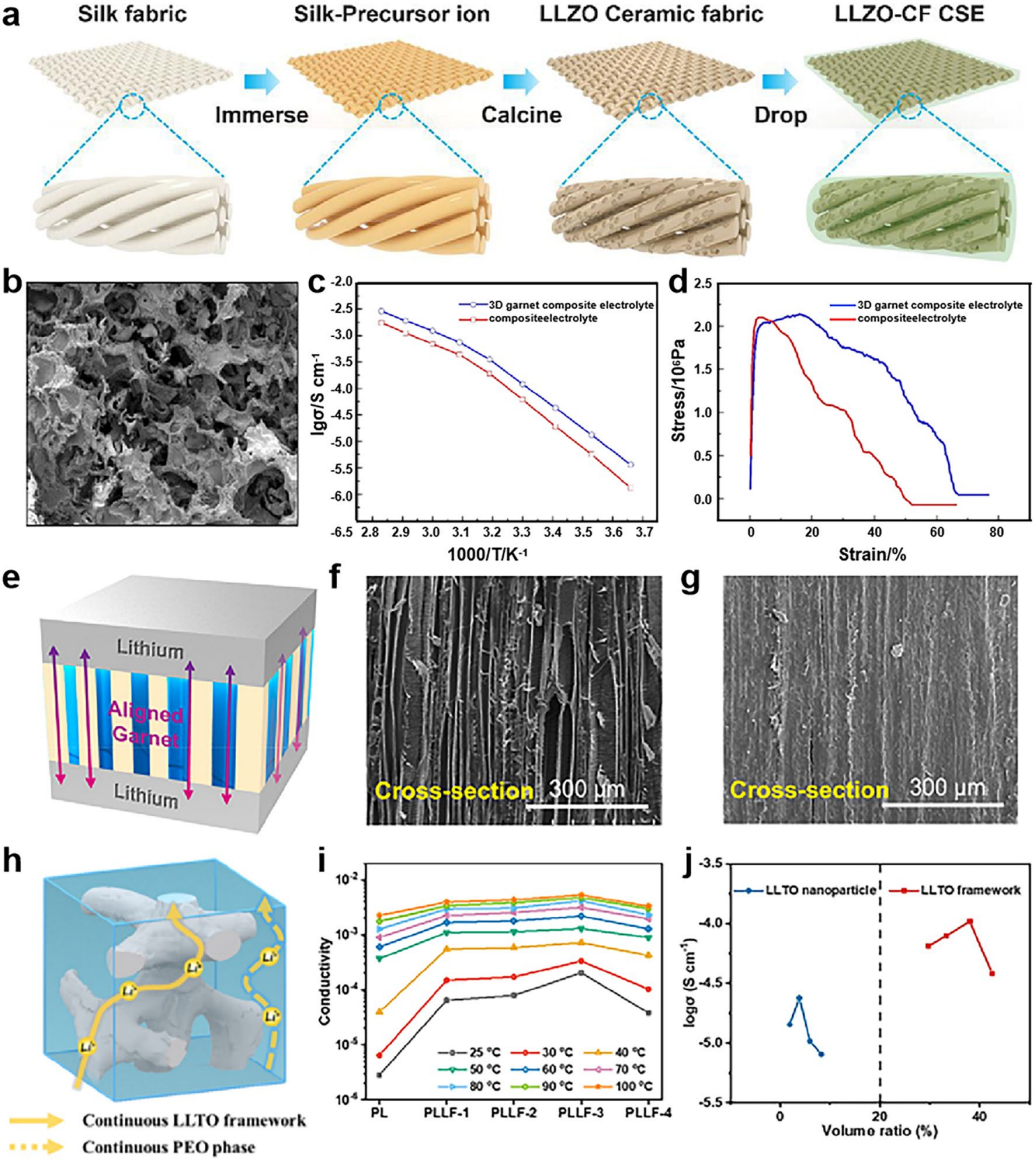
**a** Schematic of the preparation of CSEs by the templating method. Reproduced with permission [127]. Copyright 2022, Elsevier. **b** SEM image of the garnet framework. **c** Arrhenius plots of the garnet/PEO CSEs.** d** Tensile test of the CSEs. **b–d** Reproduced with permission [101]. Copyright 2019, American Chemical Society. **e** Schematic of the Li symmetric cell with garnet-wood, showing the low tortuosity and fast lithium conduction pathways. SEM images of **f** pristine wood and **g** compressed wood. **e–g** Reproduced with permission [129]. Copyright 2019, American Chemical Society. **h** Schematic representation of possible Li^+^ conduction pathway inside the PLLF electrolyte. **i** Ionic conductivity of PLLF electrolytes at different temperatures as a function of LLTO framework content. **j** The relationship between the volume ratio of inorganic components in the electrolyte and ionic conductivity at room temperature. **h–j** Reproduced with permission [133]. Copyright 2021, Elsevier

As mentioned above, constructing polymer in ceramic architectures is promising to solve the issue of ceramic particle agglomeration but in isolated forms in PEO, especially at high concentrations [101]. The templating strategy is an effective way to fabricate CSEs with continuous ceramic skeleton, which involves two major steps: (1) the formation of a porous ceramic framework assisted with a template and (2) infiltration of PEO–Li salt solution, as shown in Fig. 4a with silk fabrics as templates [126]. The frequently used templates include silk [127], cotton [128], wood [129], textile cellulose [130], polystyrene microspheres [131], cleanroom wiper [132], etc., which were infiltrated with ceramic precursors, followed by high-temperature calcination for template removal and final the formation of the ceramic framework. Subsequently, the preprepared PEO–Li salt solution is impregnated into the porous ceramic framework to obtain the CSEs by drying in a vacuum to remove the solvent.

There are several characteristics of the successful preparation of polymer in ceramic architecture CSEs via a templating strategy. First, the continuous porous structure of the template is required for forming a 3D consecutive inorganic ceramic skeleton, which is anticipated to provide better ion conduction pathways than the isolated ceramic phase in CSEs. For example, 3D continuous garnet/PEO CSEs templated by polymeric sponges exhibited higher ionic conductivity (1.2 × 10^–4^ S cm^−1^) than isolated garnet particle/PEO CSEs by the direct mixing of garnet particles and PEO–Li salt (6.4 × 10^–5^ S cm^−1^) at 30 °C (Fig. 4b, c). Meanwhile, the ion transference number (from 0.24 to 0.33), symmetrical cells (from 142 to 360 h), and mechanical strength (elongation from 5% to 22%) were also improved (Fig. 4d) [101]. Second, the pore size of the template is also important for ionic conductivity. The structural regulation of the template can tailor the porous structure of ceramics to affect their performance. For example, different pore sizes of two nylon templates (0.2 and 0.4 μm) were used to prepare LLTO skeletons, and small pore size of 0.2 μm nylon template provides a higher porosity in the reversed LLTO replica than that using 0.4 μm nylon template [133]. It was found that high porosity effectively promotes the penetration of PEO matrix into the LLTO skeleton, which is conducive to CSE fabrication. Moreover, aligned pores of ceramics provide shortened ion conduction pathways (Fig. 4e). Thus, the ionic conductivity and electrochemical performance of the cell are greatly increased [84]. At present, aligned LLZO-based CSEs were prepared by a cylindrical shape microchannel wood template (Fig. 4f, g), which exhibited the highest Li^+^ conductivity (1.8 × 10^–4^ S cm^−1^ at 25 °C) and broadened voltage window of 6.0 V [129]. However, there is currently no direct evidence comparing the advantages of aligned ceramic skeletons over twisted ones. Third, compared with ceramic in polymer CSEs (e.g., ˂ 30 wt% ceramic fillers), 3D ceramic CSEs exhibit a higher percolation threshold, such as 70 wt% LLZTO [127], 63.3 wt% LAGP [126], 63 wt% LLTO [133] and 50 wt% LLZO [128]. This is because the lower content of 3D ceramic will give rise to structural collapse upon removing the template, and sufficient 3D ceramic content could realize bicontinuous permeation networks for ion conduction. However, an excessively high content of ceramic framework with a reduced PEO phase will not be beneficial to the interfacial compatibility with the electrode, and there are also chances for the incomplete filling of PEO within 3D ceramic, failing to form continuous polymer ion channels. For example, Wang et al. [133] exhibited LLTO framework based CSEs (PLLF, Fig. 4h), which show the conductivity being the determination of mass ratios. As the mass ratio increased from 54 wt% (PLLF-1) to 63 wt% (PLLF-3), ionic conductivity increased from 6.45 × 10^–5^ to 2.04 × 10^–4^ S cm^−1^ (25 °C). However, when the LLTO skeleton mass ratio achieved 67 wt% (PLLF-4), the ionic conductivity reduced slightly (Fig. 4i). The relationship between ionic conductivity and the volume ratio of the LLTO skeleton in the CSEs follows a similar trend at room temperature (Fig. 4j).

**Fig. 5:**
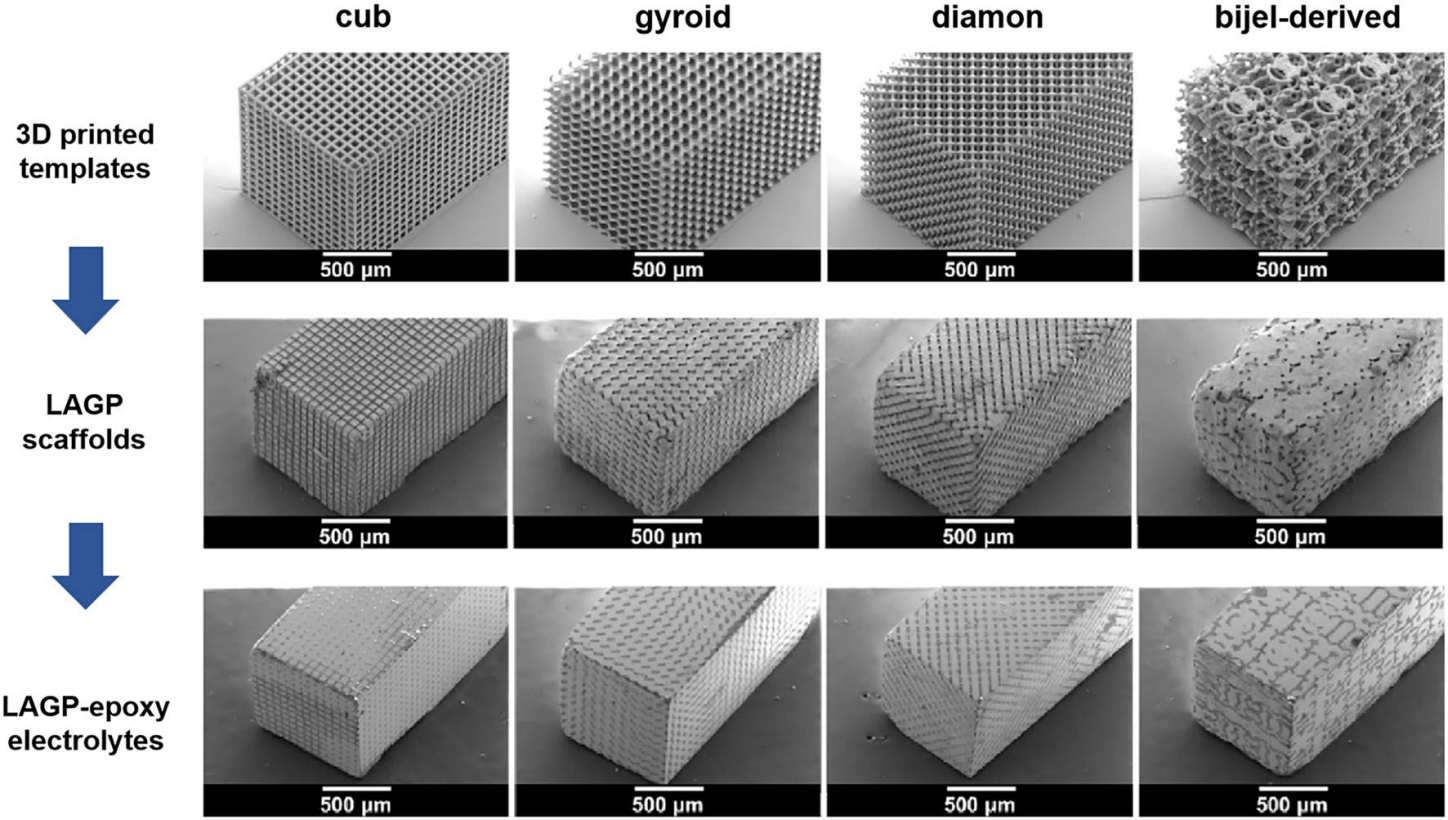
SEM images of the 3D printed templates, the structured LAGP scaffolds and the structured LAGP-epoxy electrolytes with cube, gyroid, diamond and bijel-derived (left to right) microarchitectures. Reproduced with permission [134]. Copyright 2018, Royal Society of Chemistry

Currently, the majority of templates are naturally available and cost-effective. However, the precise tailoring of the template structure is restricted, which is not beneficial for performance. Therefore, it is necessary to design and synthesize some special templates. In this case, 3D printed templates allow precise control over the ratio of ceramic-to-polymer and the microarchitecture. For example, Bruce et al. [134] prepared cube, gyroid, diamond and bijel-derived LAGP microarchitectures by 3D printing technology, which were filled with epoxy, delivering the best combination of mechanical strength and ionic conductivity (Fig. 5). The gyroid LAGP CSEs demonstrated an ionic conductivity of 1.6 × 10^–4^ S cm^−1^ at room temperature owing to decreased resistance at the grain boundaries of dense ceramic, and the mechanical properties are as high as 28% of high compressive failure strain.

Various templating strategies are used to construct continuous ceramic skeletons to provide ion conduction channels, as summarized in Table 1. However, the thickness of CSEs is relatively high, which is unfavorable due to the transport distance [53, 135], such as 120 µm (polyurethane foam template) [101], 180 µm (cleanroom wiper template) [132] and 200 µm (cellulose textile template) [130]. Based upon the above, constructing a thin film with continuous, aligned and precisely controlled 3D ceramic CSEs is the goal of using the template strategy.

**Table 1:** Performance comparison of electrolytes with different templates

Template	Composition	Thickness (µm)	LSV (V)	Ionic conductivity (S cm^−1^) (°C)	Ion transference numbers	Li symmetrical batteries	References
Silk	PEO–LiTFSI–70 wt% LLZO	127	5.1	8.89 × 10^–5^ (30)	0.49	Cycling with a current density of 100 µA cm^−2^ for 700 h at 50 °C	[127]
Nylon	PEO–LiTFSI–63 wt% LLTO	120	4.7	2.04 × 10^–4^ (25)	0.59	Cycling from 0.1 to 0.4 mA cm^−2^ for 750 h at 60 °C	[133]
Polyurethane foam	PEO–LiTFSI–40 wt% Ga–LLZO	200	5.6	1.2 × 10^–4^ (30)	0.33	Cycling with a current density of 0.4 mA cm^−2^ for 360 h at 60 °C	[101]
Cotton	PEO–LiTFSI–50 wt% LLZO	200	5.5	0.89 × 10^–4^ (25)	0.27	Cycling with a current density of 0.5 mA cm^−2^ for 500 h at 25 °C	[128]
Wood	PEO–LiTFSI–SCN-68 vol% LLZO	150	6	1.8 × 10^–4^ (25)	–	Cycling with a current density of 0.1 mA cm^−2^ for 180 h at room temperature	[129]
Cleanroom wiper	PEO–LiClO_4_–20.7 wt% LLZAO	180	5.5	2.25 × 10^–5^ (30)	0.263	Cycling with a current density of 0.3 mA cm^−2^ for 100 h at 60 °C	[132]
Ice	PEO–PEGDME–LiTFSI–63.3 wt% LAGP	100–200	–	1.67 × 10^–4^ (25)	0.56	Cycling from 0.1 to 0.3 mA cm^−2^ for 400 h at 60 °C	[126]
Cellulose textile	PEO–LiTFSI–15 vol% LLZAO	200	–	6 × 10^–5^ (25)	–	Cycling from 0.05 to 0.2 mA cm^−2^ for 550 h at 60 °C	[130]
3D print	Polypropylene–15 vol% LAGP	20		1.6 × 10^–4^ (25)	–	–	[134]
Bacterial cellulose	PEO–LiTFSI–40 wt% LLZO	70–100	6	1.12 × 10^–4^ (25)	–	–	[136]
Ice	PEO–LiClO_4_–40 vol% LATP	100	–	5.2 × 10^–5^ (25)	–	–	[137]
Polystyrene microspheres	PEO–LiTFSI–65.7 wt% LLZO	200	5.1	9.2 × 10^–5^ (25)	–	–	[131]

### Electrospinning Approach

Different from mechanical mixing for ceramic in polymer and templating strategies for polymer in ceramic architectures, the electrospinning approach is suitable for preparing both ones. For the ceramic fillers in PEO CSEs, a polymer solution (e.g., PAN [53] and Polyimide [138]) was used as a precursor to form a freestanding porous substrate, and then PEO–Li salt was cast onto/into the above fibrous films. In this process, the ceramic fillers can be either incorporated into the electrospun polymer solution (e.g., PVDF [139]) or the PEO–Li salt solution. For the polymer in ceramic architecture, the preparation process is similar to the templating strategy with the electrospun polymer (e.g., polyvinyl pyrrolidone (PVP) [140] and polyvinyl alcohol (PVA) [141]) as a template for the formation of a freestanding porous ceramic framework via calcination, followed by the infiltration of PEO–Li salt solution.

**Fig. 6:**
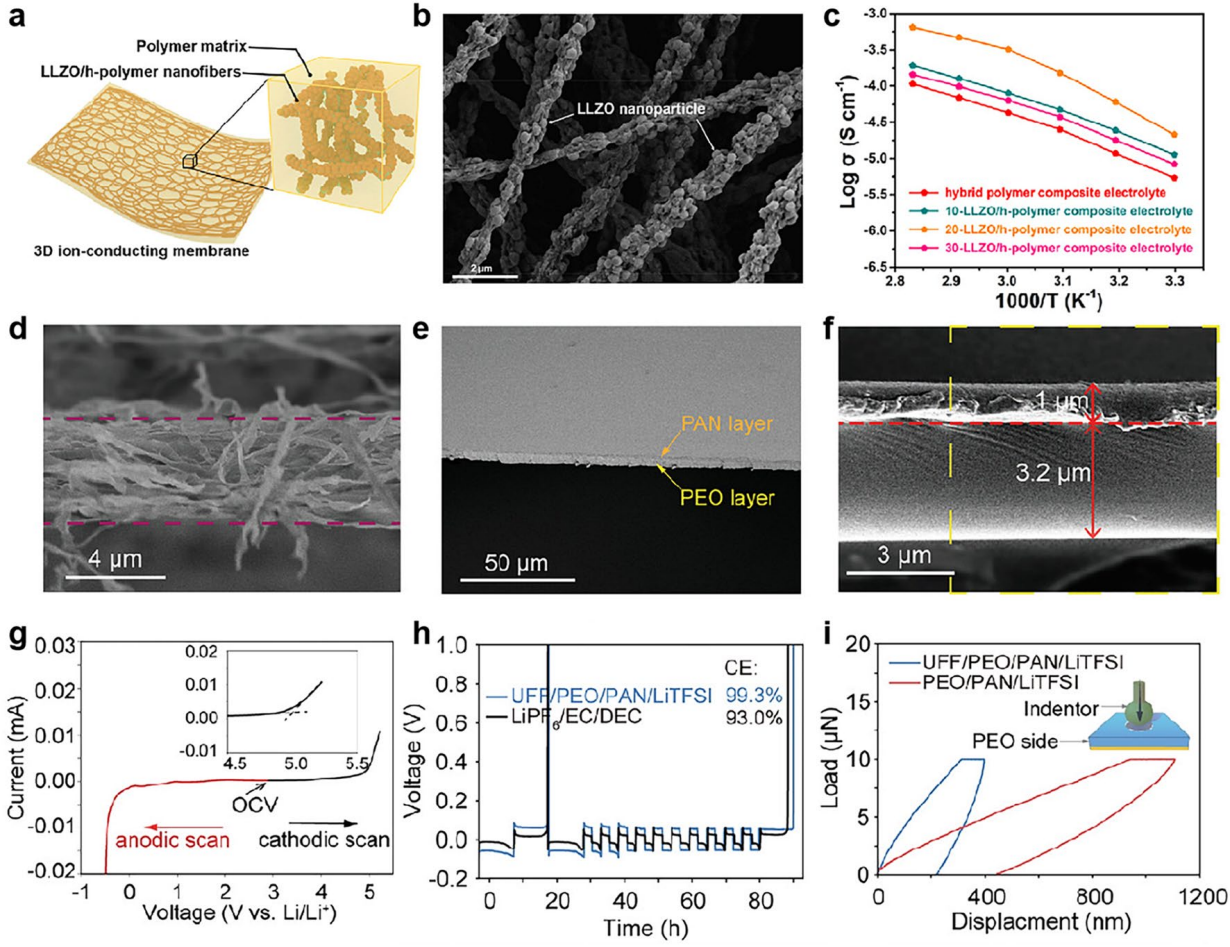
**a** Schematic diagram of CSEs with LLZO/h-polymer nanofibers. **b** SEM image of 3D ion-conducting nanofiber networks. **c** Arrhenius curves of CSEs with polymer nanofibers and CSEs with LLZO-polymer nanofibers with different LLZO contents. **a–c** Reproduced with permission [139]. Copyright 2021, American Chemical Society. **d** Cross-sectional SEM image of the as-spun UFF. **e** Cross-sectional SEM image of the double-layer UFF/PEO/PAN/LiTFSI CSEs. **f** Cross-sectional SEM enlarged image of the UFF/PEO/PAN/LiTFSI CSEs. **g** The electrochemical window of the UFF/PEO/PAN/LiTFSI electrolyte. The inset is a magnified image of the onset of the oxidation process. OCV, open circuit voltage. **h** CE measurement of Li metal (known as Aurbach CE measurement) proposed by Aurbach in Li–Cu half cells using different electrolytes in Li–Cu half cells using different electrolytes. **i** Nanoindentation test on the PEO side of the UFF/PEO/PAN/LiTFSI and PEO/PAN/LiTFSI electrolytes. **d**–**i** Reproduced with permission [141]. Copyright 2021, Wiley–VCH

The use of electrospun PAN film of the ceramic in polymer CSEs is beneficial to the stability with the cathode due to oxidation-resistant nitrile groups in PAN but is unfavorable for the ionic conductivity compared with PEO. For example, PAN films with PEO/LLZTO CSEs showed a higher electrochemical window of 4.7 V, superior to bare PEO/LLZTO CSEs (4.5 V). However, the ionic conductivity decreased from 8.18 × 10^–4^ S cm^−1^ (PEO/LLZTO CSEs) to 2.57 × 10^–4^ S cm^−1^ (PAN/PEO/LLZTO CSEs) [142]. The ionic conductivity can be tuned by the ceramic content in polymers with a percolation threshold [142–145]. For example, CSE with 20 wt% LLZO nanoparticles in electrospun PVDF films (denoted as 20-LLZO/h-polymer composite electrolyte) showed a maximum ionic conductivity of 1.05 × 10^–4^ S cm^−1^ at 50 °C (Fig. 6a–c) [139]. The polymer in ceramic CSEs was first reported by Hu et al. [146] in which a 3D LLZO ceramic network was prepared by electrospinning, followed by PEO–Li salt solution infiltration. Compared with the conventional templating strategy, it is found that the content of ceramic for forming 3D continuous networks is generally lower (10–40 wt% of total mass), such as 15 wt% LLZO [146] and 20 wt% LLTO [147]. We speculated that this might be due to the 1D fibrous networks formed by electrospinning.

The thickness of the above CSEs is related to the electrospun film thickness and the subsequent infiltration of PEO. For 3D ceramic CSEs, the thickness is reported in the range of 40–190 µm [140, 146, 148]. For example, a nanofiber network of PVP/LLTO film with a thickness of ∼ 60 μm was peeled off after spinning, and a final CSE thickness of 80–120 μm was obtained after calcination and PEO infiltration [147]. Designing much thinner CSEs is essential to achieve higher gravimetric and volumetric energy densities of full cells, which has become a current research hotspot. Interestingly, an ultrathin bilayer CSE of 4.2 µm (UFF/PEO/PAN/LiTFSI) was accomplished by filling the UFF porous scaffold (Fig. 6d) with a 1 µm PAN layer and a 3.2 µm PEO layer (Fig. 6e, f). In this design, a UFF film of 4.2 µm was prepared through the electrospinning of exfoliated vermiculite in the presence of PVA solution followed by heat treatment (Fig. 6d). The thickness of the UFF film was controlled by the electrospinning time. To fill the PEO layer, the UFF was placed on top of a viscous PEO film, which can automatically wet the UFF. After drying, the PAN solution was cast directly on the other side of the UFF film to obtain the resulting bilayer CSE. Benefiting from the bilayer polymer structure, the electrolyte achieved high compatibility with Li metal due to the PEO layer and an enlarged electrochemical window of 4.9 V due to PAN (Fig. 6g, h). Meanwhile, the stiff ceramic scaffold improves mechanical strength (Fig. 6i), delivering elastic moduli of 298 and 1072 MPa at the PEO and PAN sides, respectively, as measured by nanoindentation [141].

The macroscopic mechanical properties of ultrathin CSE films need to be cautiously considered for practical applications. There are also some thin porous polymer films in addition to electrospun polymers, such as polyimide [149, 150] and polyethylene films [135, 151], through which thin polymer solid electrolytes of ~ 10 µm can be prepared by infiltration of PEO solution. However, CSEs also function as a separator. The thinner CSEs will inevitably reduce mechanical strength, and increase the risk of membrane fracture or Li dendrite penetration, resulting in internal short circuit, battery failure, and even potential safety hazards. Currently, most CSEs are ~ 100 µm thick or more, and it remains a challenge to greatly reduce the thickness of CSEs without compromising their mechanical properties. Therefore, the relationship between the thickness and mechanical properties of CSEs needs to be systematically studied in the future.

### Gel Formation Approach

According to the process of gelatinization, the gel formation approach is divided into ceramic gels and polymer gels to obtain polymer in ceramic and ceramic in polymer architectures, respectively. For the ceramic gel-derived electrolytes, the hydrogel of the ceramic precursor is first prepared, followed by calcination to form a freestanding porous ceramic framework and finally PEO–Li salt solution infiltration. Polymer gel-based electrolytes can be readily prepared by one-pot polymerization of the monomer in the presence of ceramic fillers.

**Fig. 7:**
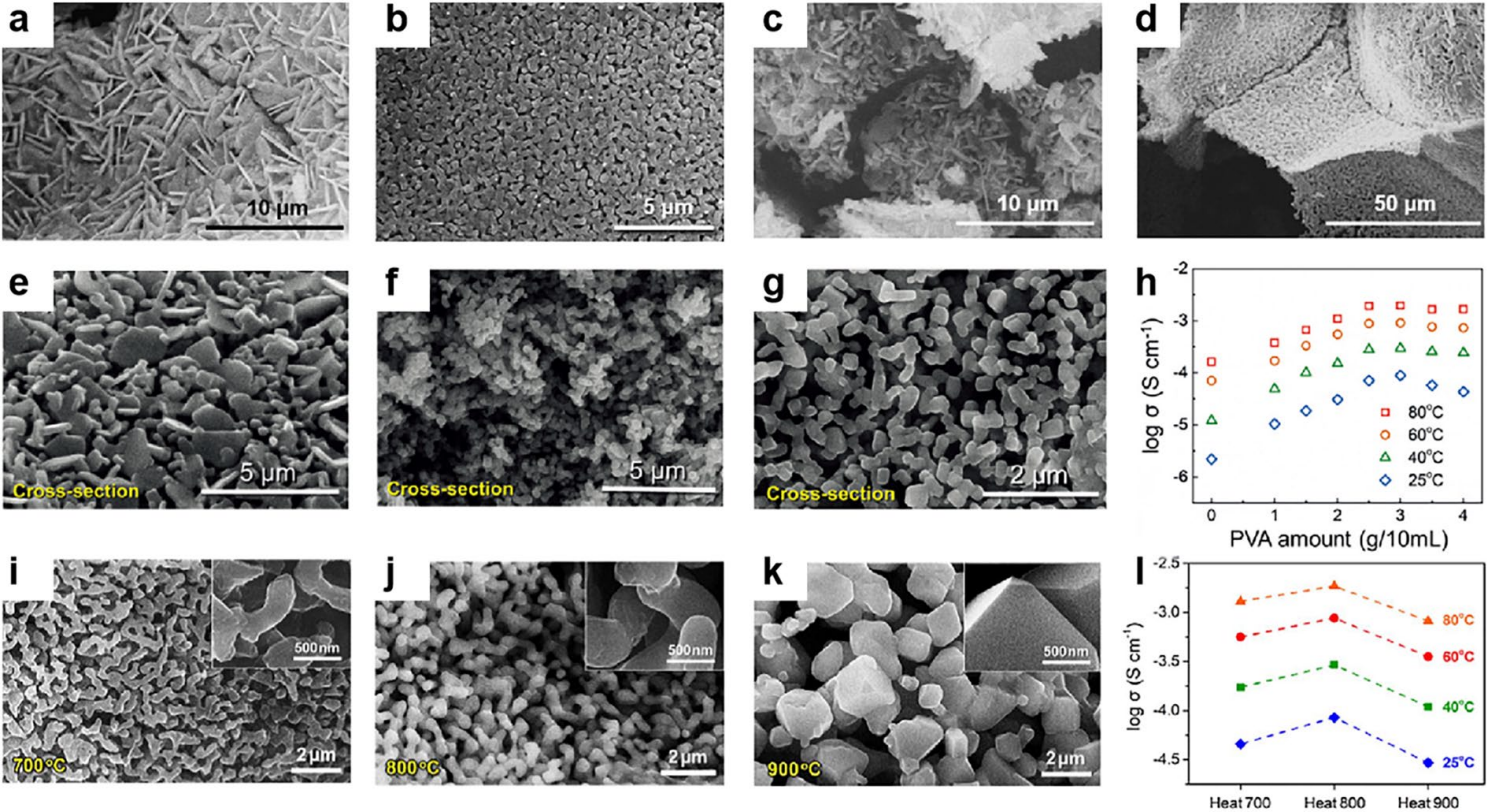
SEM images of the LLTO framework with different LLTO amounts of **a, b** 1 mmol and **c, d** 3 mmol. The surface morphologies of LLTO frameworks after heat treatment at 800 °C with different amounts of PVA: **e** 0.75 g, **f** 1.5 g, and **g** 3.0 g. **h** The conductivity of LLTO CSEs with different PVA amounts from 0 (pure PEO) to 4 g. **a**–**h** Reproduced with permission [152]. Copyright 2018, Wiley–VCH. SEM images of 3D garnet frameworks heat treated at **i** 700 °C, **j** 800 °C, and **k** 900 °C for 2 h. The insets show high-magnification SEM images of 3D garnet frameworks. **l** Conductivity of 3D-CSEs with garnet frameworks heat treated at 700 °C (heat 700), 800 °C (heat 800) and 900 °C (heat 900). **i**–**l** Reproduced with permission [153]. Copyright 2018, Elsevier

The concentrations of ceramic precursor salts, cross-linker and binder have an effect on the morphology of the ceramic gels and then the ionic conductivity. For example, by controlling the content of LLTO ceramic precursor salts and the crosslinker glutaraldehyde (Fig. 7a–d), the LLTO frameworks can exhibit nanoplate and dense particle morphology. More importantly, the binder amount can regulate the continuity of 3D ceramics with percolated structure both at the surface and inside of skeletons. When the content of binder PVA varied from 0.75 to 3.0 g (Fig. 7e–g), it was shown that 3.0 g PVA derived ceramic skeleton revealed a well-percolated structure (Fig. 7g). Correspondingly, the ionic conductivity increased to 8.8 × 10^–5^ S cm^−1^. However, when further increasing the PVA to 4 g, the ionic conductivity decreased slightly (Fig. 7h) [152]. Moreover, the morphology of the ceramic is also influenced by the heat treatment temperature. For example, the 3D garnet framework was thick branches porous, thicker branch porous and loosely connected microparticle structure when heating at 700, 800, and 900 °C, respectively (Fig. 7i–k). The results showed that the CSEs with optimized morphology and 3D interconnected structure (800 °C) exhibited the highest conductivity of 8.5 × 10^–5^ S cm^−1^ at room temperature (Fig. 7l) [153].

**Fig. 8:**
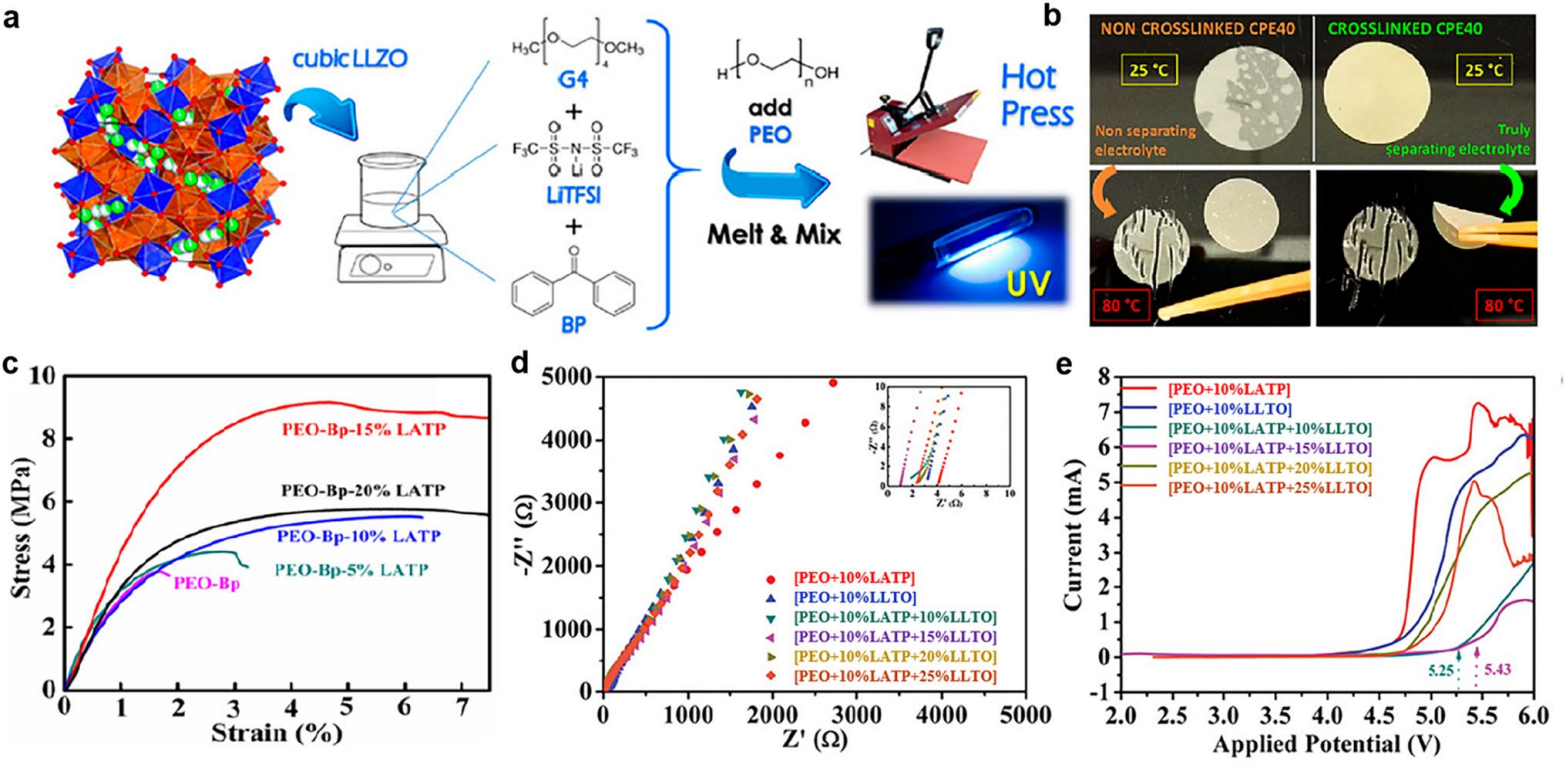
**a** Schematic illustrations of the preparation process for CSE membranes via UV irradiation. **b** The difference in terms of mechanical integrity between non-cross-linked and cross-linked CSEs after impedance test at thermal stress. **a, b** Reproduced with permission [154]. Copyright 2019, American Chemical Society. **c** Stress–strain curves for PEO-Bp and PEO-Bp-LATP membranes. Reproduced with permission [156]. **d** Impedance-spectra and **e** linear sweep voltammetry (LSV) curves of polymer gel CSEs. **d-e** Reproduced with permission [155]. Copyright 2019, Elsevier

Polymer gel CSEs are essentially quasi-solid-state electrolytes, which are usually prepared by curing PEO monomer, Li-salt and ceramic fillers in the presence of photo/thermal initiator (e.g., benzophenone), as shown in Fig. 8a with ultraviolet irradiation (UV) polymerization. Firstly, cross-linked CSEs enable thermal stability even at relatively high temperatures due to polymer networks. For example, cross-linked CSEs exhibit well mechanical integrity after impedance test at 80 °C, avoiding short circuits of battery, while non-cross-linked CSEs lost mechanical integrity (Fig. 8b) [154]. Secondly, ceramic fillers are used in CSEs to improve mechanical properties. All polymer gel CSE membranes with different content LATP (5, 10, 15 and 20 wt%) demonstrated the enhancement of tensile strength, compared with pure polymer gel electrolyte (Fig. 8c). Moreover, the type of ceramic conductor affects the ionic conductivity and electrochemical properties of gel electrolytes. For example, polymer gel-based CSEs with 10 wt% LATP showed a conductivity of 2.54 × 10^−4^ S cm^−1^ higher than that of polymer gel-based CSEs with 10 wt% LLTO (2.81 × 10^−5^ S cm^−1^) at 25 °C. Interestingly, dual ceramic polymer gel-based CSEs with 10 wt% LATP and 15 wt% LLTO exhibited higher ionic conductivity of 9.87 × 10^−3^ S cm^−1^ at 25 °C, faster Li^+^ transference number of 0.82 and wider electrochemical stability window of 5.43 V (Fig. 8d, e) as compared with a single ceramic gel based CSEs. This is because of UV cross-linked and well-dispersion of ceramic [155].

In addition, polymer gel electrolytes keep the merits of liquid electrolyte with high room temperature ionic conductivity (~ 10^−3^ S cm^−1^) and Li^+^ transport number (~ 0.8) as well as endowing with improved safety and flexibility. Unfortunately, polymer gel CSEs absorb a large number of organic solvents easily leading to thermal runaway [156, 157]. More importantly, polymer gel CSEs have a higher thickness (˃ 120 µm), thus increasing the ion conduction distance, which is not conducive to the high energy density of solid-state batteries [151].

### Other Approaches

In addition to the above well-established strategies, there are other useful and interesting strategies for the preparation of PEO/ceramic CSEs, which will be briefly covered in this section. For example, 3D porous ceramic skeletons were prepared by using pore-forming agents, e.g., SeS_2_ [158] and graphite [38]. Moreover, high-temperature rapid reactive sintering can promote the surface diffusion of grains for neck growth and limit coarsening, accurately controlling the densification and the desired porous structure. 3D LLZTO porous scaffolds by rapid reactive sintering demonstrated good ionic conductivity (~ 1.9 × 10^–4^ S cm^−1^ at room temperature) [159]. Furthermore, ultrathin CSE films were also fabricated by in situ polymerization with ceramic fillers on Li anodes. For example, a thin PEGMA/LAGP CSE of 8.5 µm was prepared by in situ copolymerization of PEGMEMA monomer in the presence of LAGP on a lithium anode, and the thickness of CSEs was controlled by scraping the mixed slurry, giving a prospective synergy of flexible-rigid property and interface compatible CSEs/lithium integration [160]. Likewise, other polymers can be also in situ polymerized in the presence of filler such as the ring-opening reaction of 1,3-dioxolane [161–163], polymerization of ethylene carbonate (EC) [164], and PEGMEA polymerization [38].

To summarize, the above preparation methods enable the design of a variety of polymer/ceramic CSE architectures with rapid progress. However, each of these methods has its strengths and limitations. One can combine concepts and tools from different strategies to develop CSEs with simultaneous high ionic conductivity and Li-ion transfer number, superior mechanical strength, flexibility and interfacial compatibility with the cathode/anode. In particular, the interface compatibility both inside the CSEs (e.g., ceramic/polymer interfaces) and between CSEs/electrodes is critical to determine the performance, and thus, control over the interface compatibility is insightfully discussed in the following [165–168].

## Interfacial Engineering

Surface modification of ceramic and constructing chemical bonds between ceramic/polymer are used for promoting smooth Li^+^ transportation at ceramic/polymer interfaces to the same goal of solving the issue of ceramic/polymer interface compatibility. Meanwhile, the interface of CSEs/electrodes can also be specially designed according to the properties (such as reactivity under different potentials) of the cathode or anode.

### Surface Modification of Ceramic

Introducing a coating layer on the surface of ceramic fillers is a promising strategy for increasing the interfacial compatibility of CSEs with ceramic in polymer architectures. The coating layer used includes polyethylene glycol (PEG) [35], polydopamine (PDA) [123] and ionic liquid [169], lithium polyacrylate [122], which can interact with ceramic via electrostatic adsorption or chemical bonding.

**Fig. 9:**
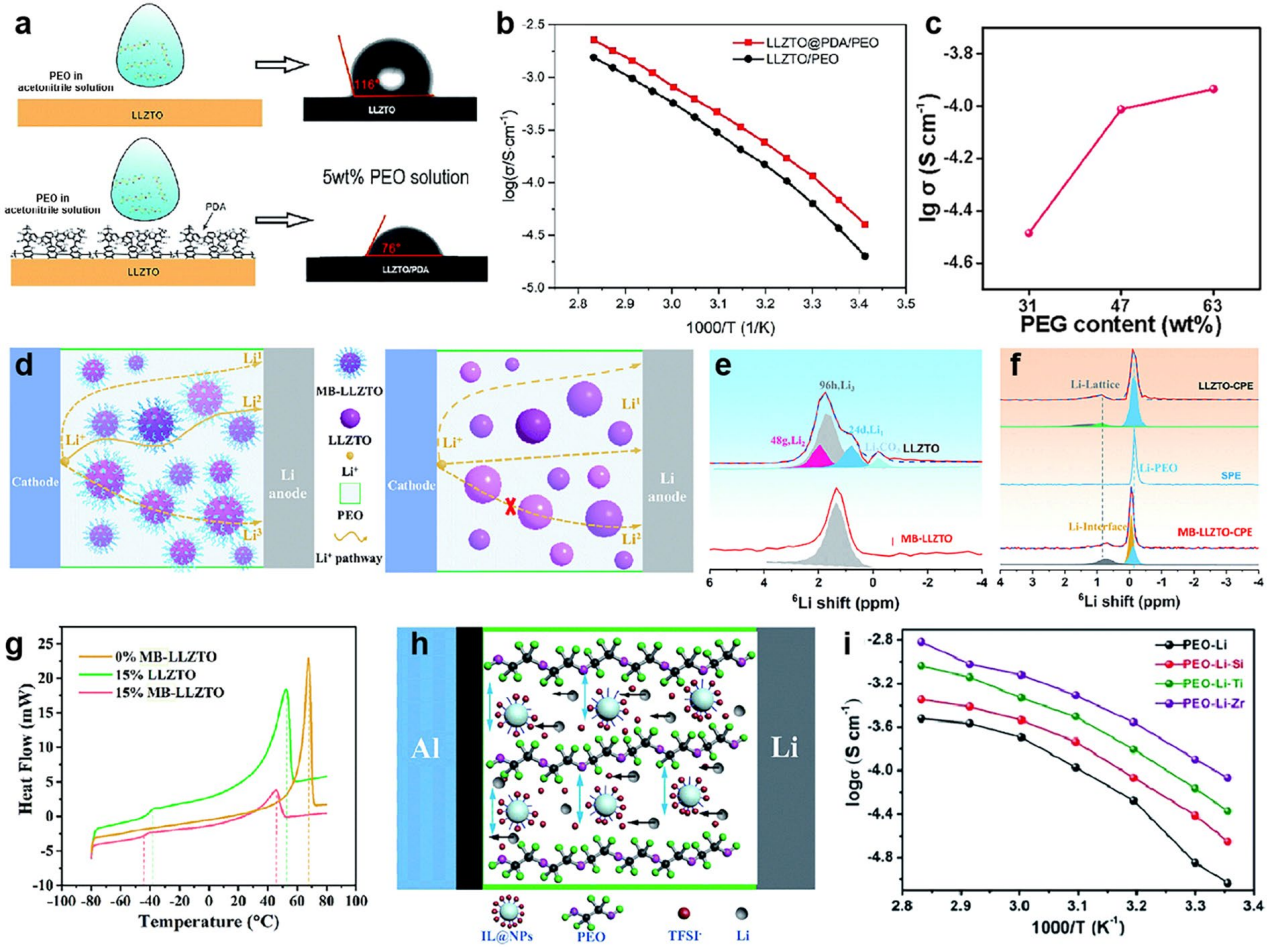
**a** Schematic of dopamine polymerized on the surface of LLZTO particles to form a polydopamine coating layer and contact angle between the PEO solution (dissolved in acetonitrile) and pristine LLZTO or LLZTO@PDA. **b** Arrhenius plots of PEO/LLZTO@PDA and PEO/LLZTO CSEs. **a, b** Reproduced with permission [123]. Copyright 2019, Royal Society of Chemistry. **c** Ionic conductivity of electrolyte membranes with different PEG contents. Reproduced with permission [80]. Copyright 2022, Elsevier. **d** Diagrams of the Li^+^ diffusion pathway in the MB-LLZTO CSEs and LLZTO CSEs. **e** ^6^Li NMR of LLZTO and MB-LLZTO materials. **f** ^6^Li direct polarization NMR spectra and assignment results for the LLZTO and MB-LLZTO CSEs. The blue area represents Li in PEO, the orange area represents the interfacial Li, and the gray and green areas correspond to the LLZTO lattice in **e**, **g** DSC thermograms of electrolytes with different coating layers. **d–g** Reproduced with permission [98]. Copyright 2019, Royal Society of Chemistry. **h** Schematic illustration of ionic liquid grafted oxide nanoparticles (IL@NPs). **i** The ionic conductivity of CSEs with different IL@NP fillers at different temperatures. **h-i** Reproduced with permission [169]. Copyright 2017, Royal Society of Chemistry. (Color figure online)

There are some criteria for the coating layer. First, the coating layer should have a similar surface energy to PEO for increasing the surface affinity between ceramic and polymer, greatly providing good compatibility and promoting Li^+^ transportation at ceramic/polymer interfaces [94, 170]. For example, PDA-coated LLZTO showed a lower contact angle of 76°, whereas pristine LLZTO showed a higher contact angle of 116° with PEO solution (Fig. 9a). Thus, the CSEs with PDA@LLZTO fillers demonstrated a higher ionic conductivity of 1.15 × 10^–4^ S cm^−1^ than CSEs with bare LLZTO (6.34 × 10^–5^ S cm^−1^) at 30 °C (Fig. 9b) [123]. Similarly, ionic liquid ([BMIM]TF_2_N) and PEG have also been used for coating ceramics, showing enhanced ionic conductivity [171]. Second, a suitable coating layer is necessary because an excess content of layers such as PEG or ionic liquid is detrimental to the mechanical properties [171]. For example, the CSEs with PEG showed a notable increase in ionic conductivity when elevating the PEG content. However, excessive PEG oligomer had a negative effect on the tensile strength of the membrane (Fig. 9c) [80]. Last but not least, for some ceramic fillers with high electrical conductivity like MXene, the selection of a coating layer (generally electrical insulation) not only helps for dispersion but also could reduce the electrical conductivity of CSEs. This is because the high electrical conductivity of CSEs could give rise to dendrite formation [172]. For example, coating mesoporous silica nanosheets on MXene fillers can reduce the electrical conductivity from 1.4 × 10^3^ to 2.3 × 10^–5^ S cm^−1^ [87].

The surface modification of ceramic can also alter the ion diffusion pathway from PEO to ceramic surfaces (Fig. 9d). A changed peak in the ^6^Li NMR spectrum of molecular brush (MB)–LLZTO demonstrated that MB altered the Li^+^ environment in the garnet (Fig. 9e). The quantified result of ^6^Li spectrum of MB–LLZTO CSEs suggested more Li on the surface than in PEO and LLZTO lattices (Fig. 9f) [98]. Moreover, the modified ceramic can also reduce PEO crystallization and weaken the interaction between PEO and Li^+^ as well as the interaction among various ions [35]. The introduced MB MB–LLZTO LLZTO reduced the crystalline area of the polymer, and further enhanced the ionic conductivity of CSEs (3.11 × 10^–4^ S cm^−1^), which is higher than that of pristine LLZTO CSEs (9.16 × 10^–5^ S cm^−1^) at 45 °C (Fig. 9g) [98]. Furthermore, surface-functionalized ceramic particles can enlarge the Li^+^ pathway in PEO chains, and the introduced anion can reduce the interaction between the polymer and Li^+^, thereby accelerating ion migration (Fig. 9h) [169]. For instance, grafting 1-methyl-3-trimethoxysilane imidazolium chloride (ILCl) to ZrO_2_, TiO_2_ and SiO_2_ were applied to CSEs. Compared with IL@TiO_2_ CSEs (1.3 × 10^–4^ S cm^−1^) and IL@SiO_2_ CSEs (7.15 × 10^–5^ S cm^−1^), the electrolyte with IL@ZrO_2_ displayed a higher ionic conductivity of 2.32 × 10^–4^ S cm^−1^ at 37 °C owing to the stronger coordination of ZrO_2_ nanoparticles and oxygen atoms in PEO chains (Fig. 9i) [169].

In addition, surface modification of the ceramic can also enhance the interface compatibility of the electrolyte/electrode, giving firmer binding and better contact. For instance, Li symmetric cells with PDA@LLZTO/PEO CSEs exhibited a lower interfacial resistance of 667 Ω cm^2^ than that of LLZTO/PEO CSEs (1367 Ω cm^2^) at 20 °C due to the surface transforming to super-lithiophilic after PDA coating [123]. Similarly, the CSEs composed of PEO and LLZTO modified with Si–R (3-glycidyloxypropyl)trimethoxysilane) layers also have decreased interfacial resistance by more than four magnitudes [173]. Various modified ceramics were used for CSEs that not only exhibit higher ionic conductivity and lower interfacial resistance but also possess a wider electrochemical stability window and better tensile strength, as illustrated in Table 2. However, systematic investigation on the type and thickness of the modified layer is still lacking, and future endeavors are suggested to optimize the performance.

**Table 2:** Performance comparison of electrolytes with different surface modifiers

Modifier	Composition	Ionic conductivity (S cm^−1^) (°C)	LSV (V)	Li symmetrical batteries	References
Mesoporous	PPO–LiTFSI–2 wt% Ti_3_C_2_	7 × 10^–10^ (25)	–	–	[87]
SiO_2_	PPO–LiTFSI–2 wt% Ti_3_C_2_@mSiO_2_	4.6 × 10^–4^ (25)	4.3	Cycling with a current density of 0.2 mA cm^−2^ for 500 h at room temperature	
PDA	PPO–LiTFSI–80 wt% LLZTO	6.34 × 10^–5^ (30)	4.5	Cycling with a current density of 0.2 mA cm^−2^ for 78 h at 50 °C	[123]
	PPO–LiTFSI–80 wt% LLZTO@PDA	1.15 × 10^–4^ (30)	4.8	Cycling with a current density of 0.2 mA cm^−2^ for 400 h at 50 °C	
IL	PEO–LiTFSI–20 wt% ZrO_2_	1 × 10^–4^ (70)	–	–	[169]
	PEO–LiTFSI–20 wt% ZrO_2_@IL	2.32 × 10^–4^ (37)	4.9	–	
HBPAE	PEO–7.5 wt% TiO_2_	–	–	–	[94]
	PEO–15 wt% TiO_2_@HBPAE	3.2 × 10^–3^ (35)	–	–	
Si–R	PEO-LiTFSI–30 wt% LZTO	1 × 10^–3^ (–)	–	–	[173]
	PEO–LiTFSI–30 wt% LLZTO@P	5 × 10^–3^ (–)	–	–	
	PEO–LiTFSI–30 wt% LLZTO@P + Si–R	9 × 10^–3^ (–)	–	–	
IL	PEO–LiTFSI–15 vol% LLZTO	1.7 × 10^–5^ (20)	4.6	–	[171]
	PEO–LiTFSI–15 vol% LLZTO@IL	2.2 × 10^–4^ (20)	4.85	Cycling with a current density of 0.5 mA cm^−2^ for 7500 h at 30 °C	
MB	PEO–LiTFSI–LLZTO	–	–	–	[98]
	PEO–LiTFSI–15 wt% LLZTO@MB	3.11 × 10^–4^ (45)	4.5	–	

### Constructing Chemical Bonds

**Fig. 10:**
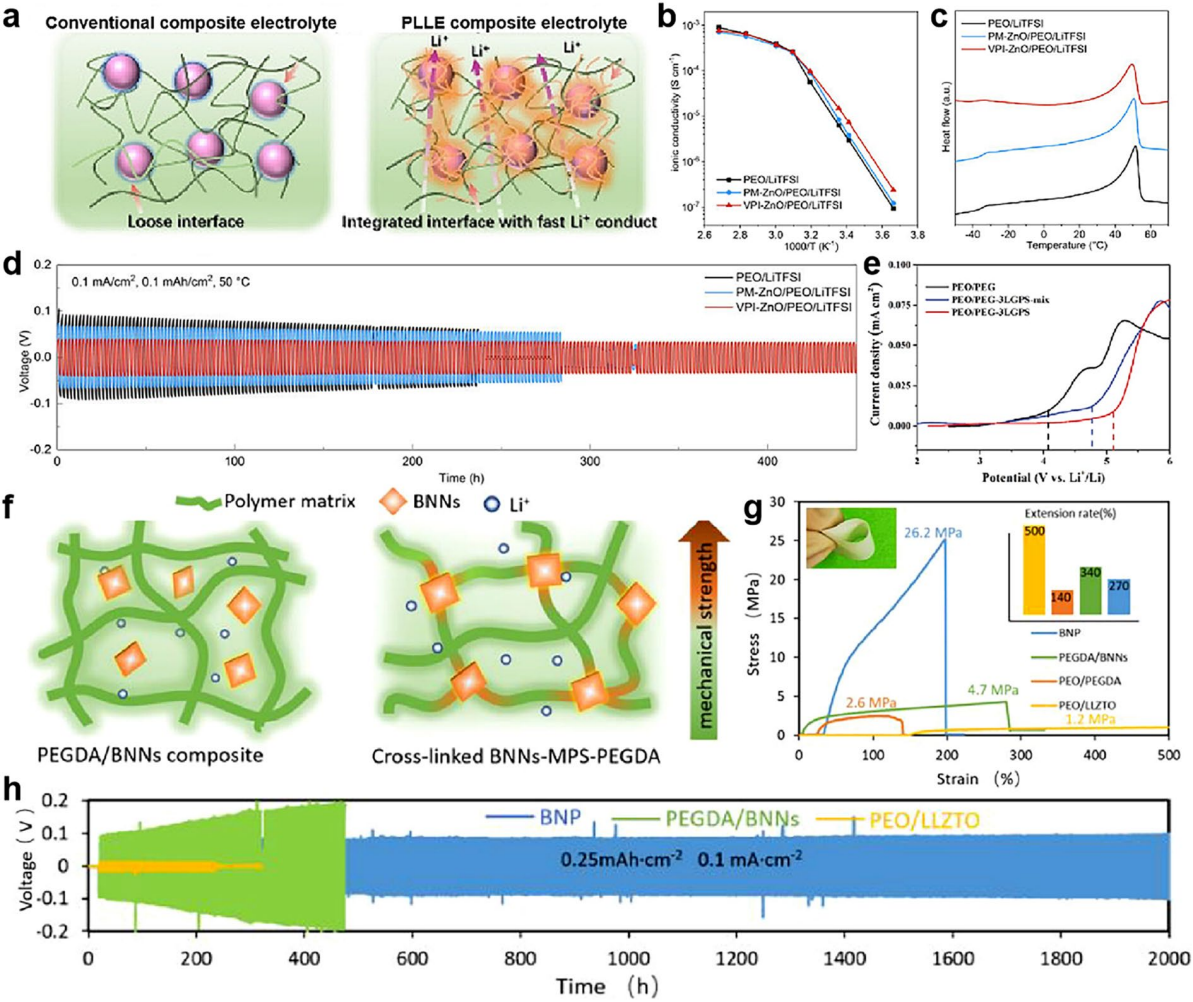
**a** Schematic figures of the synergistic effect of the components in CSEs. Reproduced with permission [97]. Copyright 2021, Wiley–VCH. **b** The relationship between ionic conductivity and temperature for each electrolyte. **c** DSC profiles of each electrolyte. **d** Long-term galvanostatic cycling of Li–Li symmetric cells. **b-d** Reproduced with permission [180]. Copyright 2021, Elsevier. **e** LSV curves of each electrolyte at room temperature. Reproduced with permission [179]. Copyright 2020, Wiley–VCH. **f** Schematic diagram of the structure of PEGDA/BNNs and BNP. **g** Stress–strain curves of different polymer electrolytes. **h** Long-cycle performance of Li symmetrical cells. **f–h** Reproduced with permission [86]. Copyright 2021, Elsevier

In addition to the above-mentioned surface coating, theoretically speaking, constructing chemical bonds can not only ensure the uniform dispersion of ceramic in CSEs for improving ionic conductivity but also increase the mechanical strength of CSEs by using ceramic as a crosslinker [174]. The in situ chemical grafting is frequently used to construct chemical bonding between ceramic fillers and polymer matrix, including in situ hydrolysis [175–178], ring-opening reactions [97], silane coupling [179] and vapor phase infiltration (VPI) chemical incorporation [180], for forming an interpenetrating polymer-ceramic network, providing dense and uniform interface Li^+^ transport channels, as seen in Fig. 10a with the intermolecular interaction of EC and LLZTO in PEO [97].

Ceramic particles uniformly disperse in CSEs by in situ hydrolysis of ceramic precursors, further lowering the crystallinity of PEO. For example, the crystallinity of in situ SiO_2_ based CSE is 19.1%, while the crystallinity of ex situ SiO_2_ CSE prepared by mechanical mixing is 20%. Correspondingly, the endothermic peak of ex situ SiO_2_ CSE appears at 49.9 °C, higher than that of in situ SiO_2_ CSE (43.8 °C). The in situ SiO_2_-based CSE had improved ionic conductivity of 1.8 × 10^–4^ S cm^−1^ as compared with ex situ SiO_2_ CSE (7.9 × 10^–5^ S cm^−1^) at room temperature [76]. Benefiting from lower crystallinity of VPI–ZnO CSE at relatively low temperatures (< 50 °C), VPI–ZnO CSE possessed higher ionic conductivity of 1.5 × 10^–5^ S cm^−1^ at 25 °C, compared with physical mixing (PM)–ZnO CSEs (Fig. 10b). Nevertheless, the ionic conductivity of VPI–ZnO CSEs was only marginally improved (Fig. 10b) due to the similar crystallinities of two samples at a temperature of nearing or higher than the melting points of PEO (Fig. 10c) [180]. Moreover, the modified PEO/ceramic interface ensures a fast Li^+^ conduction channel and reduces Li^+^ accumulation on the interface of the electrode/electrolyte, thus regulating Li^+^ deposition at the lithium anode [97]. For example, Li symmetry batteries with VPI–ZnO CSEs exhibited a low voltage polarization of approximately 40 mV, and the cycle life of the battery was prolonged to 450 h. While the symmetry batteries with PM–ZnO CSEs showed an increased overpotential to ~ 70 mV, only extending the cycle life to 284 h (Fig. 10d) [180]. Not only that, but electrochemical stability also greatly improved. For example, the coupled-LGPS CSEs start to decompose at about 5.1 V, while the mixed-LGPS CSE is approximately 4.8 V (Fig. 10e) [179].

More importantly, the mechanical strength of CSEs greatly improved due to form an interconnected network, which could effectively inhibit the growth of lithium dendrites (Fig. 10f). For example, the CSEs composed of 2D boron nitride nanosheets (BNNs) and poly(ethylene glycol)diacrylate (PEGDA) by coupled using a silane coupling agent and exhibited an ultrahigh mechanical strength (> 26.2 MPa), higher than that of uncoupled BNN CSEs (4.70 MPa, Fig. 10g). The Li symmetrical cells with coupled-BNNs also presented more uniform lithium plating/stripping, and lower overpotentials (Fig. 10h) [86]. Moreover, organic crosslinkers affect mechanical properties of CSEs. For example, the elongation and tensile strength of PEO/LiTFSI/EC CSEs decreased with adding EC, but greatly increased with adding LLZTO particles. Interestingly, the CSEs composed of PEO and LLZTO via ring-opening reaction of EC exhibited the best ionic conductivity of 1.43 × 10^–3^ S cm^−1^ at 25 °C, which is approximately equal to the ionic conductivity of a commercial liquid electrolyte (10^–3^ ~ 10^–2^ S cm^−1^ at room temperature). However, the thickness of the electrolyte is not mentioned [97].

The CSEs improved interface compatibility between polymer and ceramic by constructing chemical bonds, thus increasing ionic conductivity and mechanical properties, extending the long-cycle performance of Li symmetrical cells.

### Interface Optimization of Electrode/CSEs

Surface modification and constructing chemical bonds are utilized to resolve the issue of interface incompatibility of polymer/ceramic, and the ionic conductivity has been greatly improved, which is no longer the main bottleneck for the development of CSEs. Currently, the greatest challenge comes from the incomplete contact interface of the electrode/electrolyte that will prevent Li^+^ transport through the interface and only from the point-to-point intimate contact sites, resulting in high interface resistance [181, 182]. The large interfacial resistance leads to a slow Li^+^ transmission rate and uneven current distribution during battery operation. Moreover, volumetric changes of active materials and side reactions caused by the incompatibility of the electrolyte/electrode during battery operation also severely limit their performance [183].

**Fig. 11:**
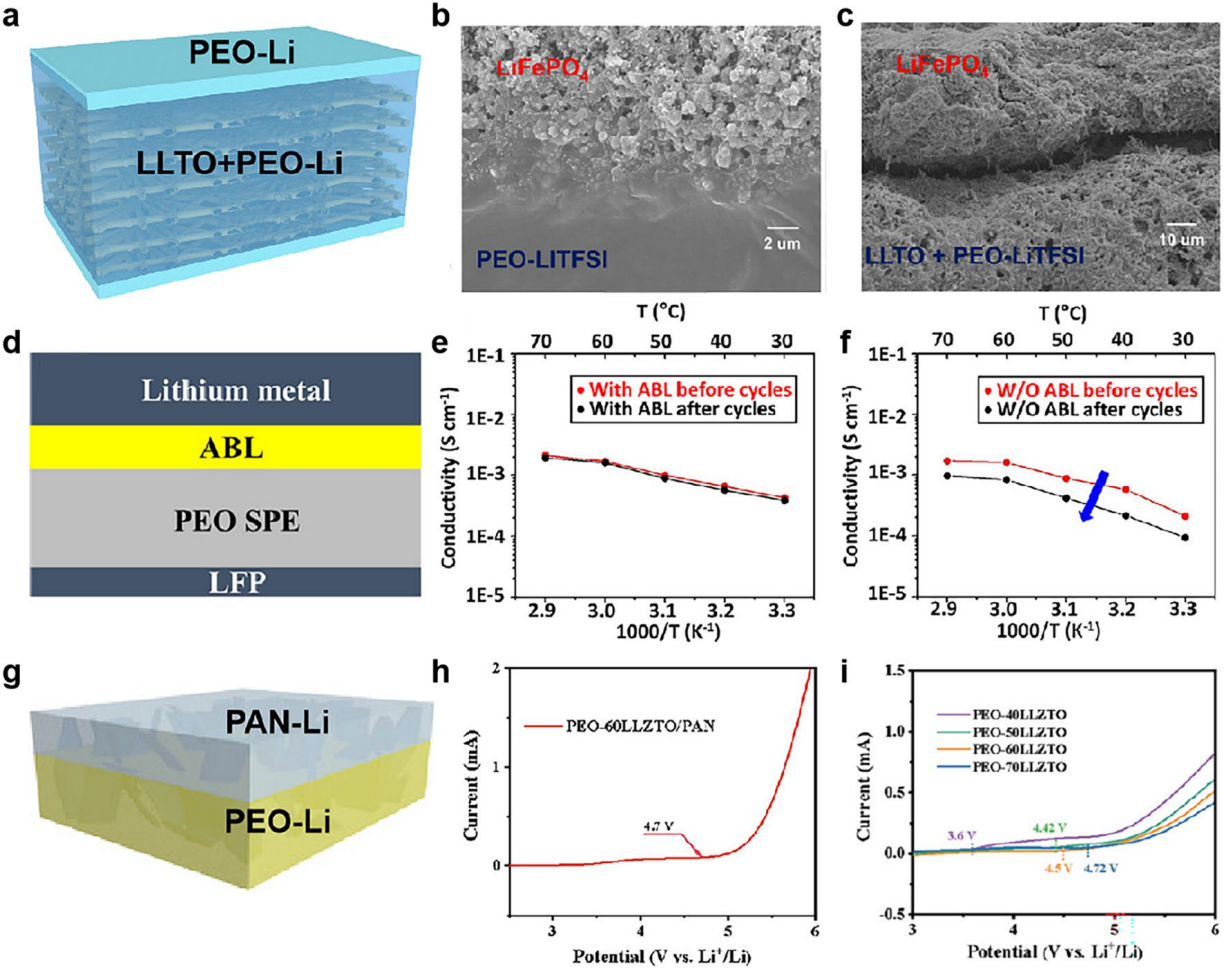
**a** Schematic of the PEO/PEO–LLTO/PEO CSEs. **b** Cross-sectional SEM images of the PEO–LiTFSI–LLTO//LiFePO_4_ and **c** PEO–LiTFSI–LLTO–O//LiFePO_4_ interfaces. **a**–**c** Reproduced with permission [183]. Copyright 2019, American Chemical Society. **d** Schematic of the interface contact between the Li metal anode and electrolyte with the ABL. Arrhenius plots of the conductivities of full batteries. **e** Li//ABL/electrolyte//LiFePO_4_ and **f** Li//electrolyte//LiFePO_4_ before and after 150 cycles. **d**–**f** Reproduced with permission [186]. Copyright 2019, American Chemical Society. **g** Structural characterization of the bilayer UFF/PEO/PAN/LiTFSI. Reproduced with permission [141]. Copyright 2021, Wiley–VCH. **h** LSV profile of CSEs with PAN. **i** LSV profiles of CSEs with different contents of LLZTO (without PAN). **h, i** Reproduced with permission [142]. Copyright 2021, Wiley–VCH

The superb interface can enable low interfacial resistance and efficient Li^+^ transport, thus significantly improving battery performance. Thus, the rational design of high stability CSEs is significantly important. Changing the structure of CSEs significantly reduces interface impedance and provides more Li^+^ transport contact sites [184, 185]. For example, changing structure of PEO/garnet electrolyte forms a sandwich structure of PEO/LLZO/PEO [181]. For ceramic embedded CSEs, some hard-to-hard contact still exists originating from the exposed ceramic surface in CSEs. Incorporating a PEO layer on either side of PEO/LLTO CSEs prevented direct contact between LLTO and electrodes (Fig. 11a). From SEM images of PEO/LLTO CSEs with two PEO layers, PEO–LiTFSI was partially immersed into LiFePO_4_ cathode and achieved a tight interface contact (Fig. 11b). In contrast, the poor interface of the CSEs without being covered by the PEO layer and LiFePO_4_ cathode led to worse performance and an obvious gap between the LiFePO_4_ cathode and CSEs is clear (Fig. 11c) [183].

Moreover, to further improve interface contact between electrolyte and electrode, introducing an extra surface modification layer on the surface of CSEs or electrode (e.g., adaptive buffer layer (ABL) [186] and Li phosphorous oxynitride (LIPON) [187]) is adopted to form CSEs for better electrochemical performance in usage for application. For example, introducing an ABL on the surface of CSE (Fig. 11d) and the battery had a small change in total resistance (increase of 6%–14%, Fig. 11e). In contrast, the total resistance increased from 43% to 63% (Fig. 11f) after cycling without ABL. This shows that the ABL can effectively prevent the decrease of the overall ionic conductivity during cycling [186]. In addition, the modification can also be on the Li anode such as LIPON-modified Li anode, to improve the compatibility between PEO/LAGP CSEs and Li anode in solid-state batteries, which offered an evenly Li^+^ flux and effectively inhibited Li dendrite formation in solid-state batteries [187].

To improve the chemical compatibility of CSEs with high-voltage cathodes, an antioxidant polymer (e.g., PAN) is introduced to enlarge the electrochemical window of CSEs (Fig. 11g) [188, 189]. For example, a bilayer CSE with PAN/PEO/UFF showed improved electrochemical stability (4.9 V), compared with PEO/UFF electrolyte without a PAN layer (4.1 V) [141]. In addition, the PAN fiber networks endow PEO/LLZTO CSEs with high oxidation resistivity, accordingly an electrochemical stability window as high as 4.7 V (Fig. 11h), higher than that of the CSEs without PAN (4.5 V, Fig. 11i) [142].

The design of CSEs improved solid–solid interface compatibility, thus maintaining better interfacial contact during battery cycling, promoting the practical application of solid-state batteries. Meanwhile, the failure process of CSEs and lithium metal deposition can be visualized by multiphysics simulation [190, 191]. Therefore, the interface optimization approach combined with multiphysics simulation has promising prospects for the rational design and development of safe solid-state batteries.

## Applications of PEO/Ceramic CSEs

Based on the advantage of PEO/ceramic CSEs, such as enhanced ionic conductivity, optimized interfacial contact, high mechanical tolerance, and excellent chemical and electrochemical stability, the PEO/ceramic CSEs coupled with anode and cathode are expected to achieve high energy density and safety solid-state batteries. This section will mainly discuss the application of PEO/ceramic CSEs in solid-state lithium metal batteries (SSLMB) with transition metal oxides and sulfur cathodes.

### SSLMB with Transition Metal Oxides Cathode

Solid-state electrolytes are a pivotal part of solid-state battery, which determines the energy and power density, cycle stability, safety performance and service life to a large extent. Some PEO/ceramic CSEs have been reported with high ionic conductivity (10^–4^ ~ 10^–3^ S cm^−1^ at room temperature), especially for polymer gel-based CSEs, which is no longer the most main problem for the practical application of solid-state batteries [99, 114, 192]. However, solid–solid interface contacts between electrodes and electrolytes, unlike the wettable liquid electrolyte, will seriously affect Li^+^ transport, increasing internal resistance, and thus deteriorating the cycle and rate performance of battery. Besides, coupled cathode materials currently used are LiFePO_4_ (LFP, ˂ 4 V), LiCoO_2_ (LCO, ~ 4.3 V) and LiNi_x_Mn_y_Co_z_O_2_ (NMC, ~ 4.3 V). Among those, LFP is extensively matched with PEO/ceramic CSEs due to lower potential (˂ 4 V), as illustrated in Table 3. However, used CSEs were limited in their potential for high-voltage applications due to the narrow electrochemical window of PEO (~ 3.9 V) [193]. In addition, some factors, such as, the thickness of CSEs, the mass of lithium metal anode, the areal capacity in cathode, negative/positive capacity (N/P) ratio, are highly regarded for realizing high-energy–density solid-state lithium metal batteries.

**Table 3:** Performance comparison of various lithium metal batteries with PEO/ceramic CSEs

Cell composition	Active materials (mg cm^−2^)	Current density (mA g^−1^)	Temperature (°C)	Specific capacity (mAh g^−1^)	Areal capacity (mAh cm^−2^)	Cycle number	References
LFP/PEGMA–LiTFSI–LAGP/Li	1	85	30	123	0.123	300	[160]
LCO/PEGMA–LiTFSI–LAGP/Li	1	274	60	140.4	0.140	120	
LFP/PEO–LITFSI–LLZTO/Li	0.8	100	60	136.4	0.109	850	[80]
LFP/PEO–LITFSI–LLZO/Li	1.5	34	50	149.3	0.224	100	[127]
LFP/PEO–LITFSI–LATP–PE/Li	1.5	170	20	93	0.140	1000	[39]
LFP/PEO–LITFSI–LLZTO/Li	1.2	34	40	116.9	0.140	500	[194]
LFP/PEO–LITFSI–LLZAO/Li	1.1	17	60	160	0.176	200	[89]
NCM/PEO–LITFSI–LLZAO/Li	1.1	17	60	150	0.165	50	
LFP/PEO–LiTFSI–Mg(ClO_4_)_2_/Li	5	20	55	157	0.785	–	[46]
NCM811/PEO–LiTFSI–Mg(ClO_4_)_2_/Li	3	33	55	145	0.435	–	
LFP/PEO–LiTFSI–LLZO/Li	3.1	34	60	151.7	0.470	500	[28]
NCM622/PEO–LiTFSI–LLZO/Li	3.1	56.2	60	145.3	0.450	200	
LFP/PEO–PVDF–LiTFSI–LLZO /Li	1.5	34	50	148.8	0.223	180	[139]
LFP/PEO–LiTFSI–LLTO/Li	1	17	60	154.7	0.155	150	[133]
NCM523/PEO–LiTFSI–LLTO/Li	2	141	60	137.2	0.274	100	
NCM622/PEO–PAN–LiTFSI–UFF/Li	23	13	50	163	3.749	100	[141]
f-Al_2_O_3_@NCM523/PEO–PVDF–LiTFSI–LLZTO–OX/Li	2	27.2	55	150.6	0.301	80	[124]
NCM811/PEO–LITFSI–VPI–ZnO/Li	1.75	140	50	164.7	0.288	200	[180]
LFP/PEO–PEGDA–LITFSI–BNNs/Li	6.4	34	25	140	0.896	150	[86]
LFP/PEO–LiCF_3_SO_3_–LATP/Li	5	85	60	118.2	0.591	1000	[92]
LFP/PEO–LiClO_4_–SiO_2_/Li	1	34	55	123.5	0.124	100	[178]
LFP/MXene–mSiO_2_/ePPO/Li	2	85	25	141.8	0.284	250	[87]
LFP/PEO–LiTFSI–SN–SiO_2_/Li	2.52	85	60	159.5	0.402	70	[195]
LFP/PEO–LiTFSI–MnO/Li	1.3	85	60	143.5	0.187	300	[90]
LCO/PEO–LiTFSI–MnO/Li	4.3	27.4	25	122.4	0.526	1000	[196]
NCM622/PEO–LiTFSI–LLZTO–SN/Li	4.8	140.5	25	153.4	0.736	300	
LFP/PEO–LITFSI–LLZTO/Li	1	17	60	153.7	0.154	200	[120]
LFP/PEO–LiTFSI–LLZO/Li	1.68	85	60	158.8	0.267	70	[117]

First, better interface compatibility between electrodes and electrolytes is conductive to interface Li^+^ transport, improving cycle and rate performance of battery, which includes the interface of electrolytes/lithium anode and electrolytes/cathode. As shown in Sect. 4.3, changing the structure of hard-to-hard contact to soft-to-hard contact or introducing an extra surface modification layer can mitigate the incomplete contact interface, providing more Li^+^ transport contact sites. Meanwhile, increasing the contact area can enhance interface compatibility by constructing composite cathodes composed of the electrolyte and cathode materials [46, 53, 139]. Second, the greater the potential difference between the positive and negative electrodes, the higher the working voltage, and the higher the energy density of battery. Thus, enlarging the electrochemical stability window of CSEs to or more than 4.3 V is essential for assembling with a high voltage cathode. Introducing high oxidation-resistant ceramics (Li^+^-insulate and Li^+^ Li^+^-conduct) or organic additives (e.g., PAN, SN) is feasible. For example, casting PEO/LiTFSI/LLZTO/SN onto PAN fiber network CSEs exhibited an increased electrochemical stability window from 3.6 (PEO/LiTFSI) to 4.72 V due to the participation of SN, PAN and LLZTO [142]. As mentioned above, surface modification of ceramics and constructing chemical bonds can also further improve the electrochemical stability of CSEs. For example, graft-coupled BNNs with PEGDA CSEs exhibited a stable electrochemical window of 5.5 V, which is higher than that of the uncoupled BNNs CSE (5.0 V) [86].

**Fig. 12:**
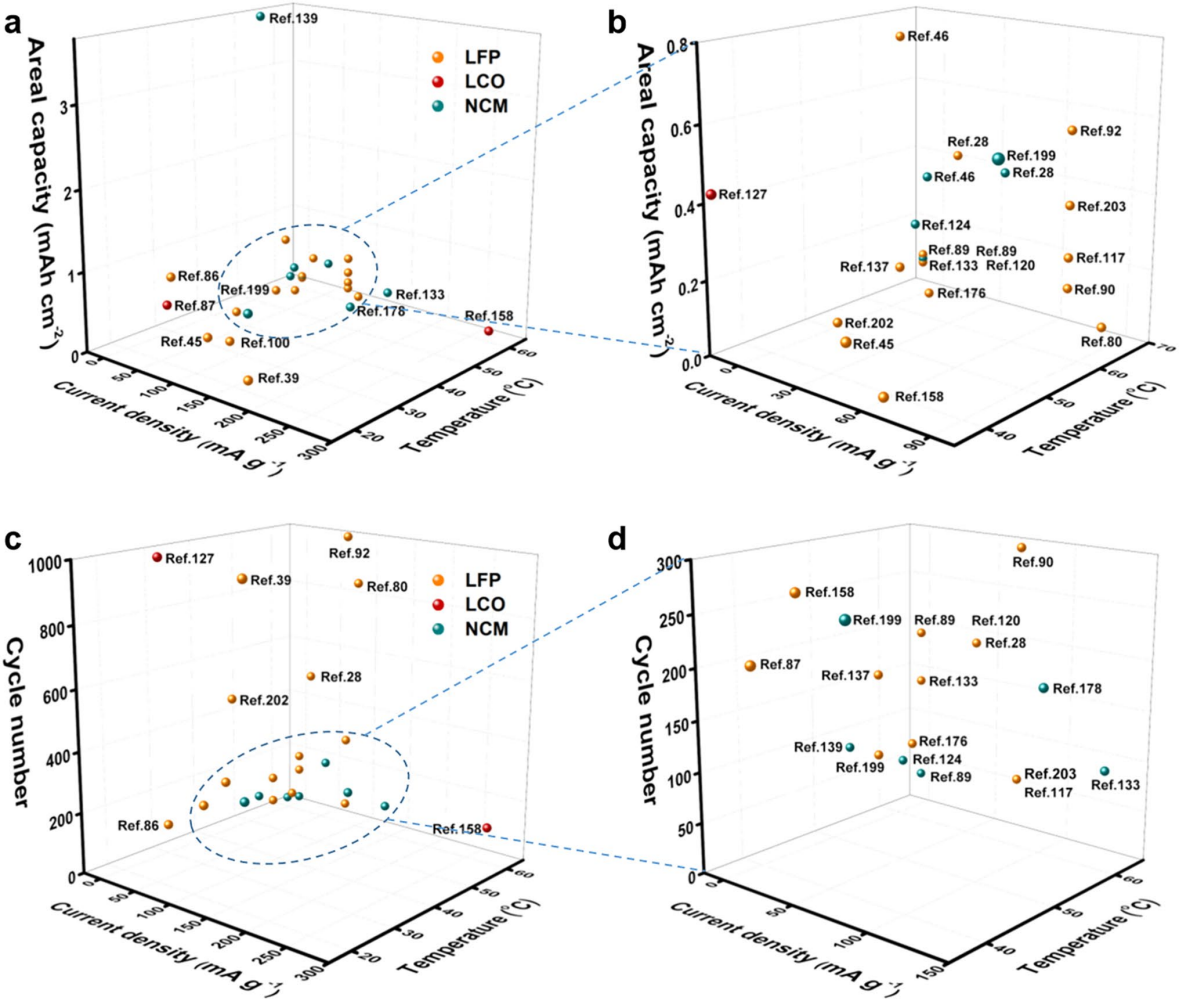
The comparison of **a**, **b** areal capacity and **c**, **d** long cycles of lithium metal batteries at different current densities and temperatures

**Fig. 13:**
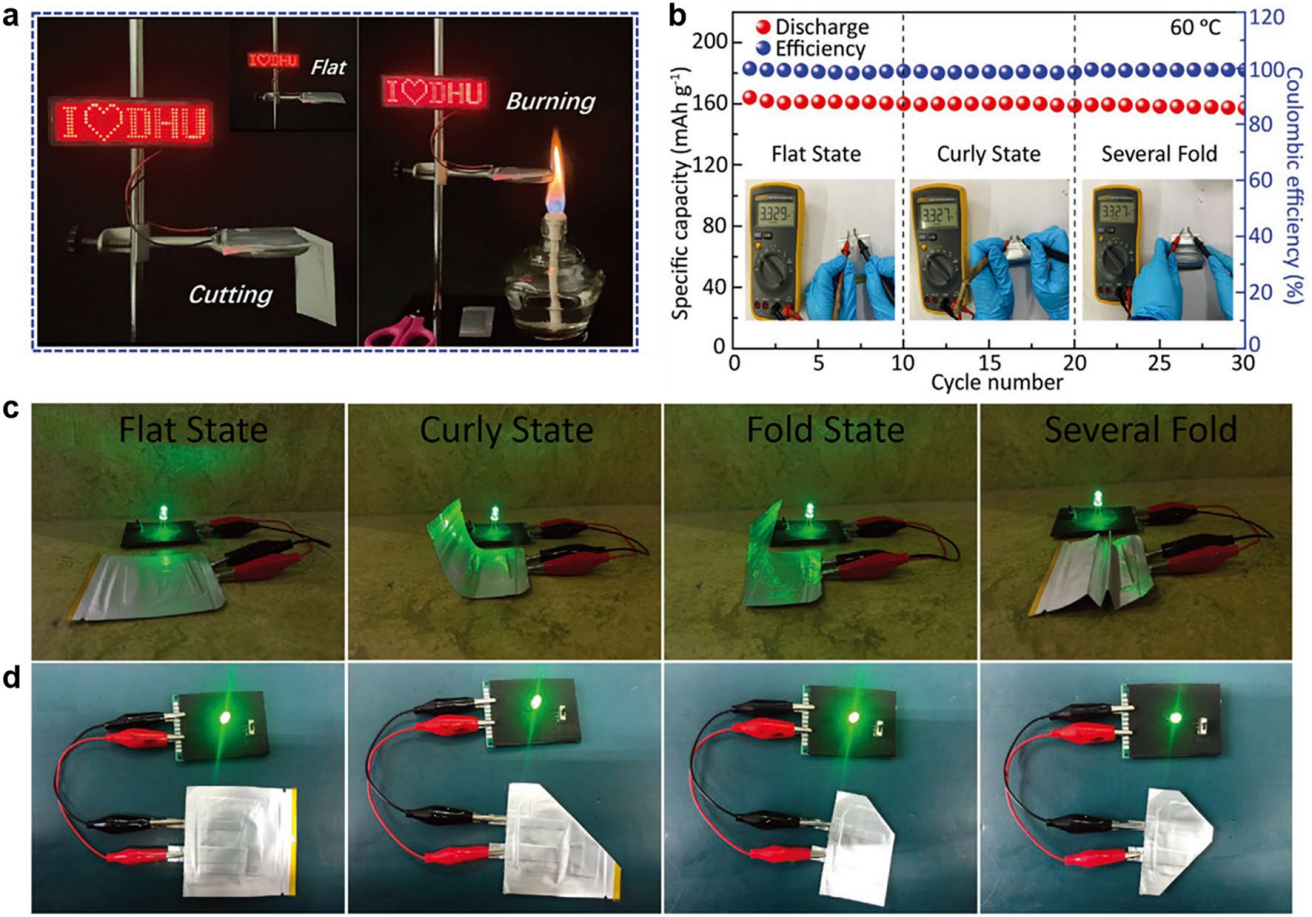
**a** Test of LCO//Li pouch cell by lighting a LED under cutting and burning. Reproduced with permission [196]. Copyright 2020, Wiley–VCH. **b** Cyclic performance of LFP//Li pouch cell under different states at 0.1C, the insets are the voltage variation images of the pouch cell before and after curling and folding. **c** Optical photographs of the flexible LFP//Li pouch cell under different states, lighting up an LED in series. **d** Safety evaluation of LFP//Li pouch cell under extreme conditions. **b**–**d** Reproduced with permission [135]. Copyright 2021, Wiley–VCH

In addition, benefiting from increasing active materials loading in cathode, the higher areal capacity furtherly enables energy density of batteries [151]. However, the excess high load will lead to failure in operating of the battery at a higher current density. For example, LFP//Li full cells with PEO/PEGDA/LiTFSI/BNNs CSEs exhibited a capacity of 0.25 mAh cm^−2^ at 0.5 C under load of 2 mg cm^−2^, when the load increased to 8 mg cm^−2^, the cell can’t operate at 0.5 C, but showed an enhanced capacity of 1.05 mAh cm^−2^ at 0.2 C [86]. Meanwhile, the full cells demonstrate better capacity at a higher test temperature (˃ melt temperature) due to the lower crystallinity of PEO. For example, under a current density of 0.5 C, LFP//Li full cells with PEGMA/LiTFSI/LAGP CSEs displayed capacities of 0.123 and 0.151 mAh cm^−2^ at 30 and 60 °C, respectively [160]. At present, the vast majority of full cells with PEO/ceramic CSEs exhibit an areal capacity of ˂ 1.1 mAh cm^−2^ (Fig. 12a, b) and operate long cycles at only higher temperatures (Fig. 12c, d). Especially, the NCM811//Li full cell with a bilayer CSE composed of PAN/UFF/PEO delivered a high capacity of 3.6 mAh cm^−2^ at 0.1 C and 50 °C due to superior active material load (17–20 mg cm^−2^), which reaches a level of commercialization (3.5 mAh cm^−2^). However, the full cell showed an uneventful cycle number of 150 and a lower capacity retention of 67% [141]. At present, the great majority of SSLMB with PEO/ceramic CSEs display a higher N/P ratio (˃ 100) due to the difficulty in achieving ultrathin lithium anode lower than 50 µm. Fortunately, an ultrathin lithium foil of 35 µm was coupled with NCM811 cathode (10.3 mg cm^−2^) in the presence of plastic-crystal-embedded elastomer CSEs (25 µm), and the cell displayed a lower N/P ratio of 3.4, a higher energy density of 410 Wh kg^−1^, which is one of the record values among SSLMB based on PEO-based CSEs, to the best of our knowledge (Table 3) [50]. To further reduce the N/P ratio, a plating 4.1 mAh cm^−2^ of Li on the Cu was assembled with superior active material load (17–20 mg cm^−2^) cathode and ultrathin PAN/UFF/PEO CSE (4.2 µm), achieving a low N/P ratio of 1.1 and a good energy density of 506 Wh kg^−1^ and 1514 Wh L^−1^ [141]. Consequently, the cycling performance of a flexible pouch cell with PEO/ceramic CSEs was tested after cutting, folding, twisting, punching and burning (Fig. 13a–d) [83, 96, 197]. For example, at different degrees of bending, the pouch cell showed continuous and stable 30 cycles (Fig. 13b) and continues to light up the LED lamp after cutting, illustrating the safety and flexibility of the pouch cell [135]. However, the pouch cells with PEO/ceramic CSEs exhibit only dozens of times continuous charge and discharge. Therefore, a superior energy density of solid-state battery is closely related to CSEs, cathode and lithium anode, and crucially, is appropriate to a pouch cell for practical application.

### SSLMB with Sulfur Cathode

Lithium-sulfur batteries are a kind of lithium metal batteries with elemental sulfur as cathode and lithium metal as anode. The working principle of lithium-sulfur batteries is as follows: lithium metal is oxidized to form Li^+^ and electrons in the discharge process. The Li^+^ moves to the sulfur cathode through electrolyte, and the electrons reach cathode through external circuit wire. Sulfur reacts with Li^+^ and electrons to form Li_2_S at the cathode with the formation of several soluble lithium polysulfide intermediates. The charging process is opposite. Lithium-sulfur batteries have a high theoretical specific capacity of 1671 mAh g^−1^ based on sulfur cathode, which is also naturally abundant, low cost, and environmentally friendly [198]. However, in addition to the problems mentioned similar with typical lithium metal batteries, they also face many notorious problems: low utilization of sulfur active material, the polysulfide shuttle effect, side reaction, safety, poor electronic/ionic conductivity of sulfur and lithium sulfide (Li_2_S), etc.

**Fig. 14:**
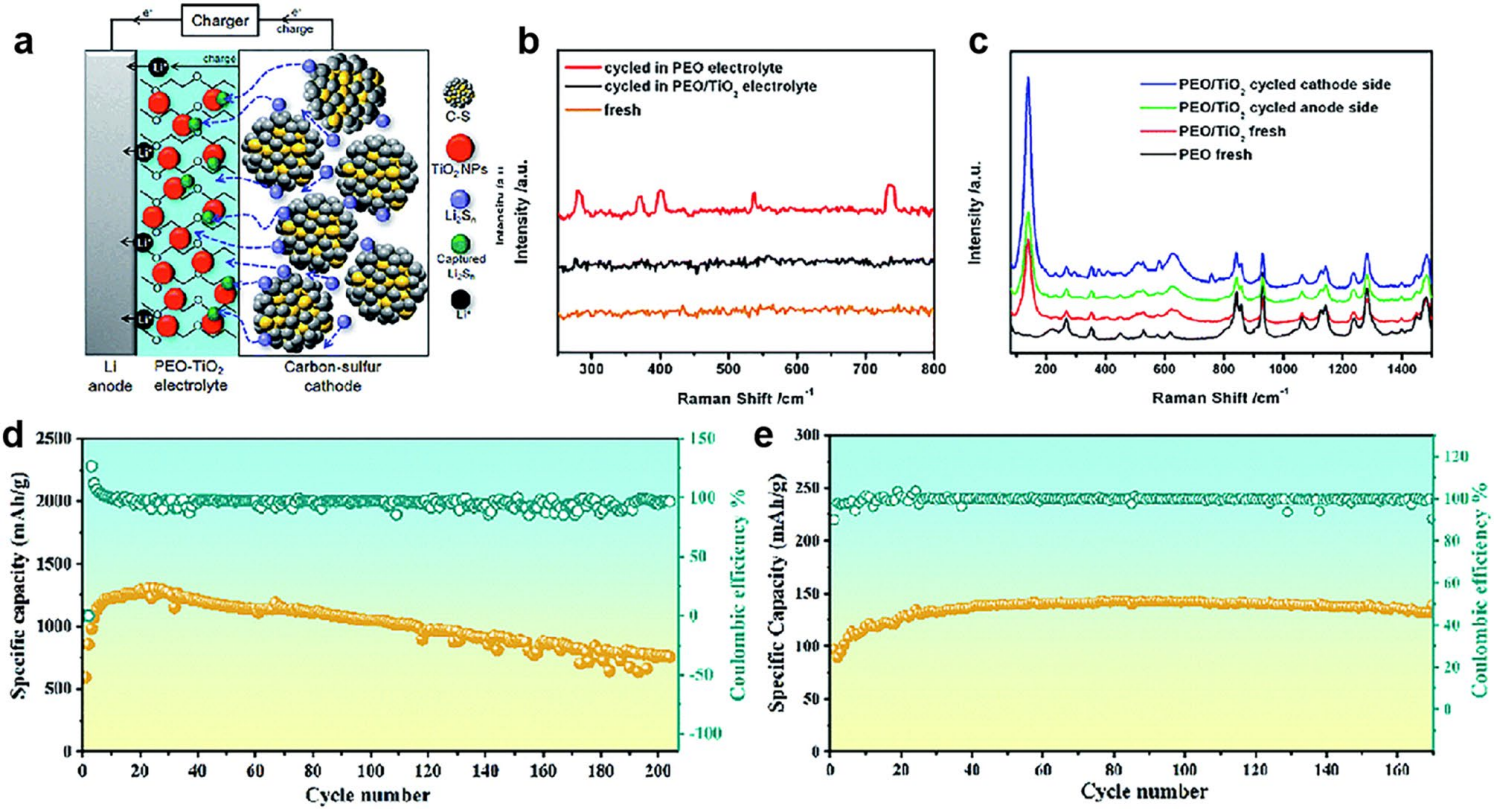
**a** Schematic illustration of the inhibition of soluble Li_2_S_*n*_ diffusion. Upon charging, Raman spectra of **b** Li anode and PEO and **c** PEO/TiO_2_ electrolytes before and after cycling. **a**–**c** Reproduced with permission [201]. Copyright 2017, Royal Society of Chemistry. **d** Discharge capacity and Coulombic efficiency of the Li–S battery based on MB-LLZTO CSE at 45 °C. **e** Discharge capacity and Coulombic efficiency of the MB–LLZTO CSE-based LFP–Li battery at 45 °C. **d**, **e** Reproduced with permission [98]. Copyright 2019, Royal Society of Chemistry

In recent years, PEO/ceramic CSEs have been widely applied in solid-state lithium-sulfur batteries (SSLSB) due to their high safety and capability to tackle the issue of sulfur cathode and Li anode [169, 199, 200]. For example, the incorporation of TiO_2_ nanoparticles into PEO can suppress the undesired shuttle effect owing to dissolved lithium polysulfides (Fig. 14a–c) [201]. The cells with TiO_2_/PEO CSEs exhibited an improved capacity retention of ~ 87% after 100 cycles, higher than that of PEO electrolytes without TiO_2_ nanoparticles (~ 38%). This is because polysulfide species are effectively withheld or trapped by the embedded TiO_2_ nanoparticles to retard the polysulfide shuttling. Benefiting from the high specific capacity of sulfur, the SSLSB with MB-LLZTO CSEs exhibited a specific capacity of ~ 1280 mAh g^−1^ and capacity retention of 60% after 220 cycles (Fig. 14d). However, the battery with LiFePO_4_ cathode exhibited a discharge capacity of only 140 mAh g^−1^ after 170 cycles (Fig. 14e) [98].

**Fig. 15:**
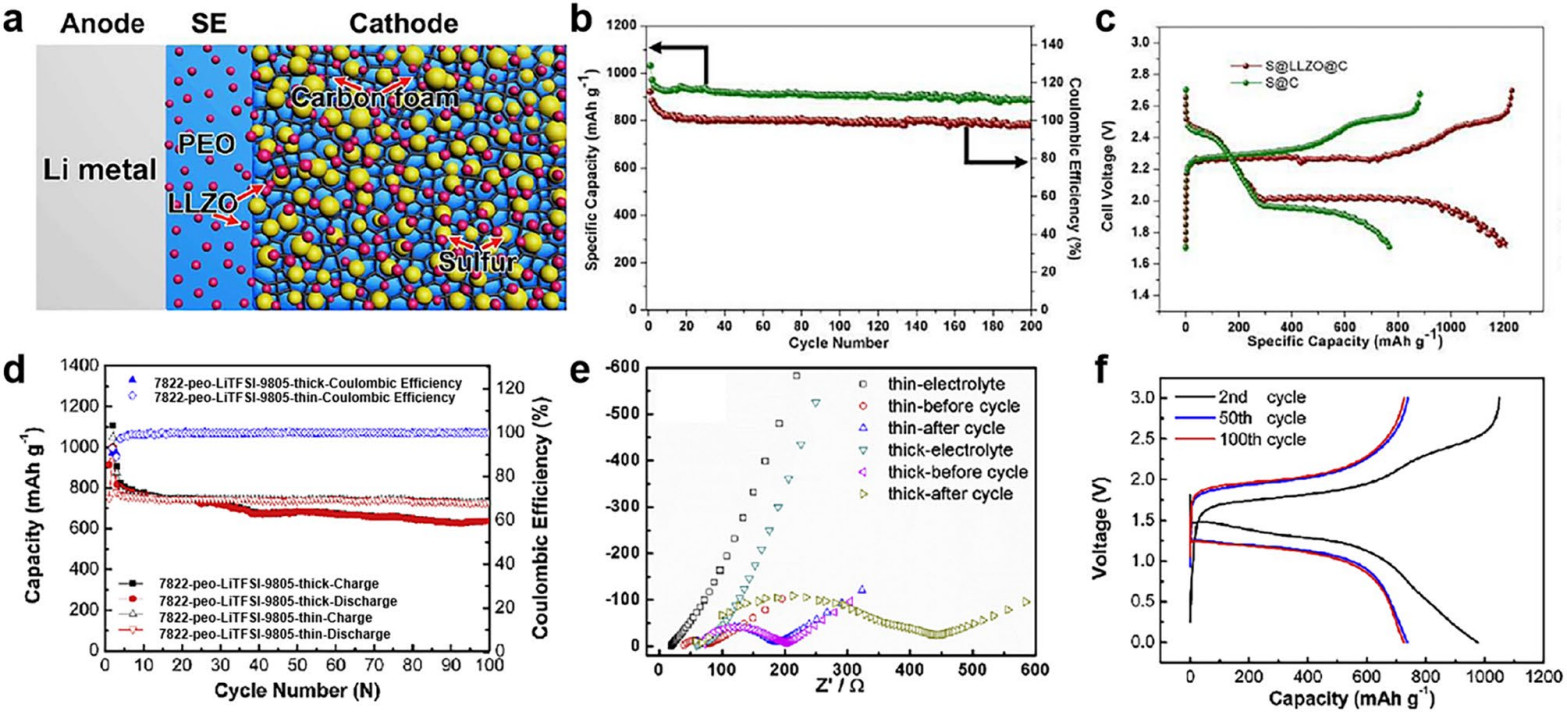
**a** Schematic illustration of an SSLSB based on LLZO nanostructures. **b** Cycling performance and Coulombic efficiency of the S@LLZO@C cathode with a current density of 0.05 mA cm^−2^ at 37 °C. **c** Typical charge/discharge curves of the S@LLZO@C and S@C cathodes with 0.1 mA cm^−2^ at 50 °C. **a**–**c** Reproduced with permission [202]. Copyright 2017, American Chemical Society. **d** Cycle performance of S-CNT//7822-peo-LiTFSI-9505//Li-In all-solid-state cells. **e** Nyquist plots for the cells using 80 or 20 mg composite electrolytes before and after the 100th cycle. **f** The charge–discharge voltage profiles at the 2nd, 50th, and 100th cycles of the cells using 20 mg 7822-peo-LiTFSI-9505 as the electrolyte layers. **d**–**f** Reproduced with permission [203]. Copyright 2020, Elsevier

Similarly, the composite cathode with the electrolyte can enhance interface compatibility by increasing the contact area [130]. For example, Cui et al. [202] prepared a composite cathode (S@LLZO@C) by a thermal diffusion method and the dispersed LLZO nanoparticles acting as the interfacial stabilizer, which delivered a specific capacity of 1210 mAh g^−1^, higher than that of the S@C cathode of 768 mAh g^−1^ (Fig. 15a, b). Moreover, larger potential hysteresis is observed in the voltage curves of the S@C cathode (Fig. 15c), indicating that the S@LLZO@C nanostructure can reduce the interface resistance to ensure high electronic and ionic conductivity simultaneously. In addition, the cells with thinner electrolytes that have a higher ionic conductance result in better cycle performance. For example, CSEs of 80 and 20 mg correspond to thicknesses of 520 ± 10 and 120 ± 5 μm, respectively. The cell with 20 mg CSE possessed a lower initial discharge capacity of 778.1 mAh g^−1^, but a higher capacity retention of 93.2% after 100th cycle, as compared with the cell with 80 mg CSE (818.6 mAh g^−1^, 77.9% (Fig. 15d). Meanwhile, the cell with 20 mg CSE exhibited lower interfacial impedances (Fig. 15e) and superior cycle performance (Fig. 15f) [203].

In addition, some challenges similar to typical lithium metal batteries, such as the high excess of lithium metal anode, the low areal capacity in sulfur cathode, high N/P ratio, high operating temperature, should be cautiously considered for realizing high-energy-density SSLSB. At present, the majority of SSLSB with PEO/ceramic CSEs did not provide N/P ratio probably due to the use of excess of lithium anode (commercial lithium disc around of 400–500 µm). Fortunately, a pressed Li foil with a thickness of 80 µm were coupled with sulfur cathode with 10.0 mg cm^−2^ of sulfur delivering a low N/P ratio of 1/1, and the cell exhibited a high areal capacity of 11.8 mAh cm^−2^ at 1 mA cm^−2^ [35], which is one of the record values among PEO/ceramic CSEs in SSLSB, to the best of our knowledge. Besides, vast majority of sulfur cathode showed a low sulfur loading (0.5–3 mg cm^−2^), and the cell delivered unsatisfactory the areal capacity (˂ 2.0 mAh cm^−2^), and the cells operated at a higher temperature (˃ 36 °C). More importantly, the pouch cells with PEO/ceramic CSEs are lacking. To our knowledge, only two cases were reported that can power LED lamps and operate for 90 cycles at 1 mA cm^−2^ [35, 98]. Therefore, optimizing CSEs, cathode and lithium anode is required for achieving an excellent energy density of SSLSB, especially for pouch cells of practical application.

## Summary and Outlook

**Fig. 16:**
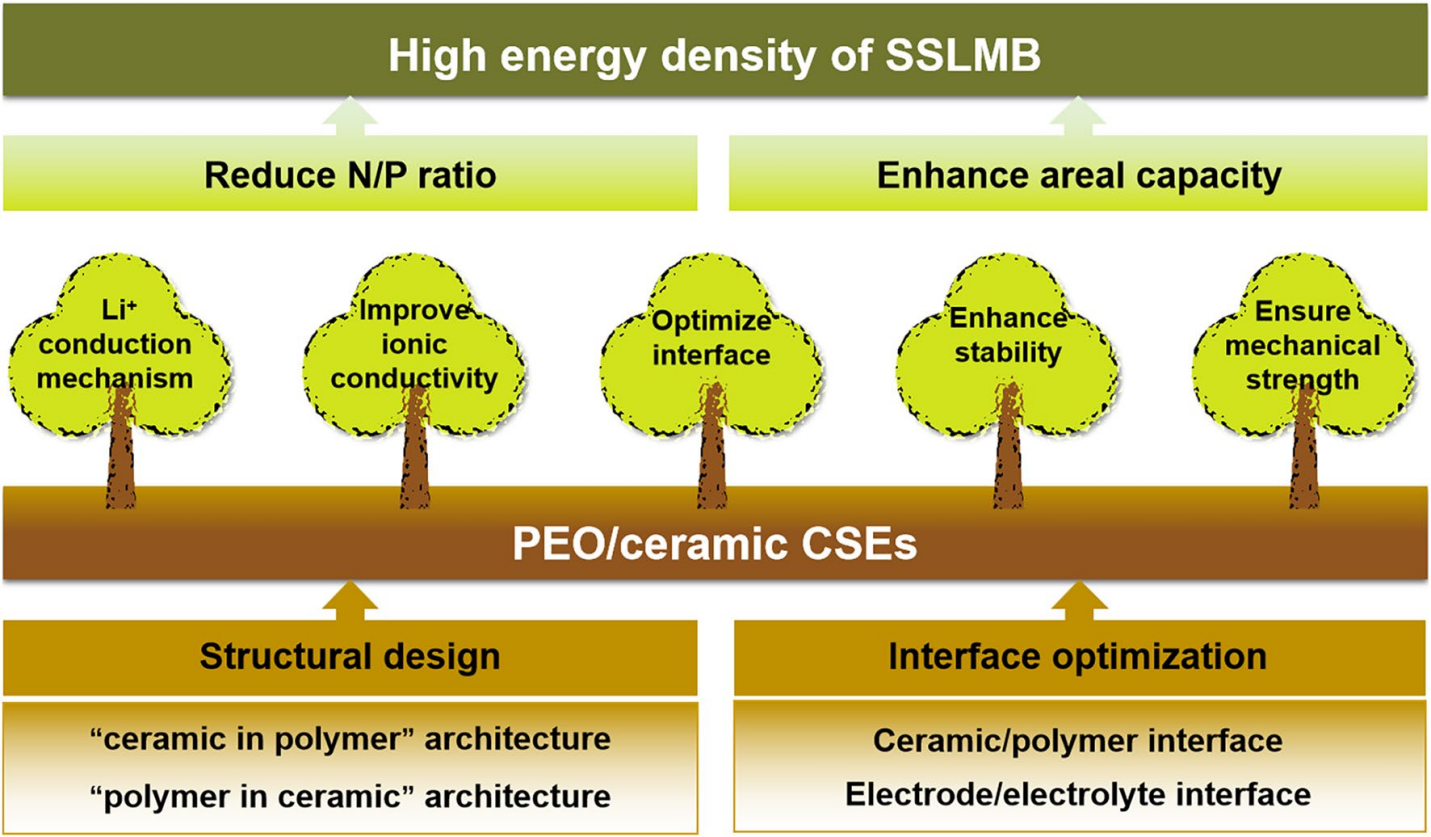
Schematic illustration of significant challenges and strategies to achieve excellent SSLMB

LMBs are considered to be a strong competitor over traditional lithium-ion batteries. However, the unsafety of using volatile and flammable organic liquid electrolytes prevents their practical applications. PEO/ceramic CSEs are promising substitutes for liquid electrolytes and have attracted wide attention in the research of energy storage systems. In this review, the critical issues of PEO/ceramic CSEs are specifically introduced. We emphasize fabricating strategies of PEO/ceramic CSEs, controlling interfacial compatibility and improving solid-state battery performance. Benefiting from the versatile preparation methods, PEO/ceramic CSEs can tackle the trade-off of ionic conductivity and mechanical properties. In addition, much effort has been made to increase interfacial compatibility, such as surface modification of ceramic particles, constructing chemical bonds and interface optimization of electrodes/CSEs. Regarding the application of full cells, we summarize solid-state lithium metal batteries with transition metal oxides and sulfur cathodes. To achieve high energy density and safety of SSLMB, the research directions are proposed in the future (Fig. 16).

For PEO/ceramic CSEs: (a) Insightfully investigate Li^+^ conduction mechanism or behavior. Theoretical calculations and advanced characterization techniques especially in situ tests are key to studying the local structural environments and dynamics of lithium ions to confirm the experimental results; (b) Improve ionic conductivity and reduce electronic conductivity at low operating temperatures. The vast majority of CSEs have an ionic conductivity of ~ 10^–4^ S cm^−1^ at room temperature, which still exhibits a gap compared with liquid electrolytes (~ 10^–2^ S cm^−1^). At the same time, Li^+^ transference number is enhanced to further clarify the ion mobility behavior; (c) Optimize interfacial contact. The described interface of CSEs mainly includes the interface of polymer/ceramic and CSEs/electrodes. The solid–solid interface seriously affects Li^+^ transport, increasing internal resistance, thus deteriorating the cycle and rate performance of battery; (d) Reduce the thickness of CSEs and shorten ion conduction distance, while ensuring mechanical strength and flexibility. Reduced mass and volume can improve energy density of solid-state batteries.

From the viewpoint of SSLMB: (a) Reduce the thickness of lithium metal anode such as down to 20 μm (5 mAh cm^−2^), which can greatly reduce N/P ratio and improve Li utilization to achieve high energy density; (b) Increase mass loading of active materials in cathode to achieve areal capacity close to or even beyond to current commercial Li-ion batteries (4 mAh cm^−2^). Reducing the N/P ratio and enhancing areal capacity are significant for achieving an energy density of SSLMB; (c) Assemble pouch cells is necessary to evaluate their potential for commercialization. A superior energy density of solid-state batteries is closely related to CSEs, cathode and lithium anode. Not only that, suitable pressures are necessary to the pouch cells when designing the structure of pouch cells.

Upcoming efforts can focus on the design of multifunctional CSEs to match ultrathin lithium metal anodes and high-load cathodes for achieving a high energy density of SSLMB (500 Wh kg^−1^). In addition, there is a strong demand for realizing large-scale production and increasing energy density and cycle stability. However, when the battery is magnified to a pouch cell, the problems mentioned above will be further magnified, which means that the cycle life of lithium metal anode with high capacity will be further shortened. Thus, precise control of the structure-performance relationship of CSEs together with lithium metal anodes and cathodes beyond the laboratory level is necessary.

## References

[1] Barreto RA (2018). Fossil fuels, alternative energy and economic growth. Econ. Model..

[2] Zhang XQ, Cheng XB, Zhang Q (2016). Nanostructured energy materials for electrochemical energy conversion and storage: a review. J. Energy Chem..

[3] Zhang Q, Suresh L, Liang QJ, Zhang YX, Yang L (2021). Emerging technologies for green energy conversion and storage. Adv. Sustain. Syst..

[4] Gao YL, Pan ZH, Sun JG, Liu ZL, Wang J (2022). High-energy batteries: beyond lithium-ion and their long road to commercialisation. Nano-Micro Lett..

[5] Xu TZ, Wang D, Li ZW, Chen ZY, Zhang JH (2022). Electrochemical proton storage: from fundamental understanding to materials to devices. Nano-Micro Lett..

[6] Blomgren GE (2017). The development and future of lithium ion batteries. J. Electrochem. Soc..

[7] Li M, Lu J, Chen ZW, Amine K (2018). 30 years of lithium-ion batteries. Adv. Mater..

[8] Grey CP, Hall DS (2020). Prospects for lithium-ion batteries and beyond-a 2030 vision. Nat. Commun..

[9] Placke T, Kloepsch R, Dühnen S, Winter M (2017). Lithium ion, lithium metal, and alternative rechargeable battery technologies: the odyssey for high energy density. J. Solid State Electr..

[10] Wang QY, Liu B, Shen YH, Wu JK, Zhao ZQ (2021). Confronting the challenges in lithium anodes for lithium metal batteries. Adv. Sci..

[11] Xu W, Wang JL, Ding F, Chen XL, Nasybulin E (2014). Lithium metal anodes for rechargeable batteries. Energy Environ. Sci..

[12] Min XQ, Xu GJ, Xie B, Guan P, Sun ML (2022). Challenges of prelithiation strategies for next generation high energy lithium-ion batteries. Energy Storage Mater..

[13] Wang JM, Ge BC, Li H, Yang M, Wang J (2021). Challenges and progresses of lithium-metal batteries. Chem. Eng. J..

[14] Wang HS, Yu ZA, Kong X, Kim SC, Boyle DT (2022). Liquid electrolyte: The nexus of practical lithium metal batteries. Joule.

[15] Tan SJ, Wang WP, Tian YF, Xin S, Guo YG (2021). Advanced electrolytes enabling safe and stable rechargeable Li-metal batteries: progress and prospects. Adv. Funct. Mater..

[16] Liu TC, Wang JL, Xu Y, Zhang YF, Wang Y (2021). Dendrite-free and stable lithium metal battery achieved by a model of stepwise lithium deposition and stripping. Nano-Micro Lett..

[17] Yu ZA, Cui Y, Bao ZN (2020). Design principles of artificial solid electrolyte interphases for lithium-metal anodes. Cell Rep. Phys. Sci..

[18] Fan XL, Wang CS (2021). High-voltage liquid electrolytes for Li batteries: progress and perspectives. Chem. Soc. Rev..

[19] Lee H, Oh P, Kim J, Cha H, Chae S (2019). Advances and prospects of sulfide all-solid-state lithium batteries via one-to-one comparison with conventional liquid lithium ion batteries. Adv. Mater..

[20] Zhang Y, Zuo TT, Popovic J, Lim K, Yin YX (2019). Towards better Li metal anodes: challenges and strategies. Mater. Today.

[21] Wu WB, Bo YY, Li DP, Liang YH, Zhang JC (2022). Safe and stable lithium metal batteries enabled by an amide-based electrolyte. Nano-Micro Lett..

[22] Ren WH, Ding CF, Fu XW, Huang Y (2021). Advanced gel polymer electrolytes for safe and durable lithium metal batteries: challenges, strategies, and perspectives. Energy Storage Mater..

[23] Li XY, Wang Y, Xi K, Yu W, Feng J (2022). Quasi-solid-state ion-conducting arrays composite electrolytes with fast ion transport vertical-aligned interfaces for all-weather practical lithium-metal batteries. Nano-Micro Lett..

[24] Yi JG, Chen L, Liu YC, Geng HX, Fan LZ (2019). High capacity and superior cyclic performances of all-solid-state lithium-sulfur batteries enabled by a high-conductivity Li_10_SnP_2_S_12_ solid electrolyte. ACS Appl. Mater. Interfaces.

[25] Lu Y, Meng XY, Alonso JA, Fernández-Díaz MT, Sun CW (2019). Effects of fluorine doping on structural and electrochemical properties of Li_6.25_Ga_0.25_La_3_Zr_2_O_12_ as electrolytes for solid-state lithium batteries. ACS Appl. Mater. Interfaces.

[26] Lin ZY, Guo XW, Yang YB, Tang MX, Wei Q (2021). Block copolymer electrolyte with adjustable functional units for solid polymer lithium metal battery. J. Energy Chem..

[27] Tian ZC, Kim D (2022). A flexible, robust, and high ion-conducting solid electrolyte membranes enabled by interpenetrated network structure for all-solid-state lithium metal battery. J. Energy Chem..

[28] Wang S, Sun QF, Peng WX, Ma Y, Zhou Y (2021). Ameliorating the interfacial issues of all-solid-state lithium metal batteries by constructing polymer/inorganic composite electrolyte. J. Energy Chem..

[29] Li S, Zhang SQ, Shen L, Liu Q, Ma JB (2020). Progress and perspective of ceramic/polymer composite solid electrolytes for lithium batteries. Adv. Sci..

[30] Zhao CT, Sun Q, Luo J, Liang JN, Liu YL (2020). 3D porous garnet/gel polymer hybrid electrolyte for safe solid-state Li–O_2_ batteries with long lifetimes. Chem. Mater..

[31] Liu XY, Li XR, Li HX, Wu HB (2018). Recent progress of hybrid solid-state electrolytes for lithium batteries. Chem. Eur. J..

[32] Wan J, Xie J, Mackanic DG, Burke W, Bao Z (2018). Status, promises, and challenges of nanocomposite solid-state electrolytes for safe and high performance lithium batteries. Mater. Today Nano.

[33] Zhang QQ, Liu K, Ding F, Liu XJ (2017). Recent advances in solid polymer electrolytes for lithium batteries. Nano Res..

[34] Ganesan SV, Mothilal KK, Selvasekarapandian S, Ganesan TK (2018). The effect of titanium dioxide nano-filler on the conductivity, morphology and thermal stability of poly(methyl methacrylate)-poly(styrene-co-acrylonitrile) based composite solid polymer electrolytes. J. Mater. Sci. Mater. Electron..

[35] Pei F, Dai SQ, Guo BF, Xie H, Zhao CW (2021). Titanium-oxo cluster reinforced gel polymer electrolyte enabling lithium-sulfur batteries with high gravimetric energy densities. Energy Environ. Sci..

[36] Zhang DC, Liu ZB, Wu YW, Ji SM, Yuan ZX (2022). In situ construction a stable protective layer in polymer electrolyte for ultralong lifespan solid-state lithium metal batteries. Adv. Sci..

[37] Fan W, Zhang XL, Li CJ, Zha SY, Wang J (2019). UV-initiated soft-tough multifunctional gel polymer electrolyte achieves stable-cycling Li-metal battery. ACS Appl. Energy Mater..

[38] Yan YY, Ju JW, Dong SM, Wang YT, Huang L (2021). In situ polymerization permeated three-dimensional Li-percolated porous oxide ceramic framework boosting all solid-state lithium metal battery. Adv. Sci..

[39] Li S, Lu JZ, Geng Z, Chen Y, Yu XQ (2022). Solid polymer electrolyte reinforced with a Li_1.3_Al_0.3_Ti_1.7_(PO_4_)_3_-coated separator for all-solid-state lithium batteries. ACS Appl. Mater. Interfaces.

[40] Chen B, Huang Z, Chen XT, Zhao YR, Xu Q (2016). A new composite solid electrolyte PEO/Li_10_GeP_2_S_12_/SN for all-solid-state lithium battery. Electrochim. Acta.

[41] Keller M, Appetecchi GB, Kim GT, Sharova V, Schneider M (2017). Electrochemical performance of a solvent-free hybrid ceramic-polymer electrolyte based on Li_7_La_3_Zr_2_O_12_ in P(EO)_15_LiTFSI. J. Power Sources.

[42] Chen WP, Duan H, Shi JL, Qian YM, Wan J (2021). Bridging interparticle Li^+^ conduction in a soft ceramic oxide electrolyte. J. Am. Chem. Soc..

[43] Huang B, Xu BY, Zhang JX, Li ZH, Huang ZY (2020). Li-ion conductivity and stability of hot-pressed LiTa_2_PO_8_ solid electrolyte for all-solid-state batteries. J. Mater. Sci..

[44] Blake AJ, Kohlmeyer RR, Hardin JO, Carmona EA, Maruyama B (2017). 3D printable ceramic-polymer electrolytes for flexible high-performance Li-ion batteries with enhanced thermal stability. Adv. Energy Mater..

[45] Yao PC, Zhu B, Zhai HW, Liao XB, Zhu YX (2018). PVDF/palygorskite nanowire composite electrolyte for 4 V rechargeable lithium batteries with high energy density. Nano Lett..

[46] Xu BY, Li XY, Yang C, Li YT, Grundish NS (2021). Interfacial chemistry enables stable cycling of all-solid-state Li metal batteries at high current densities. J. Am. Chem. Soc..

[47] Sheng OW, Hu HL, Liu TF, Ju ZJ, Lu GX (2021). Interfacial and ionic modulation of poly (ethylene oxide) electrolyte via localized iodization to enable dendrite-free lithium metal batteries. Adv. Funct. Mater..

[48] Xia Y, Wang XL, Xia XH, Xu RC, Zhang SZ (2017). A newly designed composite gel polymer electrolyte based on poly(vinylidene fluoride-hexafluoropropylene) (PVDF–HFP) for enhanced solid-state lithium-sulfur batteries. Chemistry.

[49] Yu XR, Wang LL, Ma J, Sun XW, Zhou XH (2020). Selectively wetted rigid-flexible coupling polymer electrolyte enabling superior stability and compatibility of high-voltage lithium metal batteries. Adv. Energy Mater..

[50] Lee MJ, Han JH, Lee K, Lee YJ, Kim BG (2022). Elastomeric electrolytes for high-energy solid-state lithium batteries. Nature.

[51] Zhang Y, Feng W, Zhen YC, Zhao PY, Wang XH (2022). Effects of lithium salts on PEO-based solid polymer electrolytes and their all-solid-state lithium-ion batteries. Ionics.

[52] Tong RA, Chen LH, Fan BB, Shao G, Liu RP (2021). Solvent-free process for blended PVDF–HFP/PEO and LLZTO composite solid electrolytes with enhanced mechanical and electrochemical properties for lithium metal batteries. ACS Appl. Energy Mater..

[53] Ma YX, Wan JY, Yang YF, Ye YS, Xiao X (2022). Scalable, ultrathin, and high-temperature-resistant solid polymer electrolytes for energy-dense lithium metal batteries. Adv. Energy Mater..

[54] Fenton DE, Parker JM, Wright PV (1973). Complexes of alkali metal ions with poly(ethylene oxide). Polymer.

[55] Lascaud S, Perrier M, Vallée A, Besner S, Prud’homme J (1994). Phase diagrams and conductivity behavior of poly(ethylene oxide)-molten salt rubbery electrolytes. Macromolecules.

[56] Quartarone E, Mustarelli P, Magistris A (1998). PEO-based composite polymer electrolytes. Solid State Ionics.

[57] Fan P, Liu H, Marosz V, Samuels NT, Suib SL (2021). High performance composite polymer electrolytes for lithium-ion batteries. Adv. Funct. Mater..

[58] Qiu JL, Liu XY, Chen RS, Li QH, Wang Y (2020). Enabling stable cycling of 4.2 V high-voltage all-solid-state batteries with PEO-based solid electrolyte. Adv. Funct. Mater..

[59] Nie KH, Wang XL, Qiu JL, Wang Y, Yang Q (2020). Increasing poly(ethylene oxide) stability to 4.5 V by surface coating of the cathode. ACS Energy Lett..

[60] Croce F, Appetecchi GB, Persi L, Scrosati B (1998). Nanocomposite polymer electrolytes for lithium batteries. Nature.

[61] Wu JY, Yuan LX, Zhang WX, Li Z, Xie XL (2021). Reducing the thickness of solid-state electrolyte membranes for high-energy lithium batteries. Energy Environ. Sci..

[62] Popovic J, Brandell D, Ohno S, Hatzel KB, Zheng J (2021). Polymer-based hybrid battery electrolytes: theoretical insights, recent advances and challenges. J. Mater. Chem. A.

[63] Wang XE, Kerr R, Chen FF, Goujon N, Pringle JM (2020). Toward high-energy-density lithium metal batteries: opportunities and challenges for solid organic electrolytes. Adv. Mater..

[64] Du LL, Zhang B, Wang XF, Dong CH, Mai LQ (2023). 3D frameworks in composite polymer electrolytes: synthesis, mechanisms, and applications. Chem. Eng. J..

[65] Meng N, Zhu XG, Lian F (2022). Particles in composite polymer electrolyte for solid-state lithium batteries: a review. Particuology.

[66] Shen ZC, Cheng YF, Sun SH, Ke X, Liu LY (2021). The critical role of inorganic nanofillers in solid polymer composite electrolyte for Li^+^ transportation. Carbon Energy.

[67] Feng J, Wang L, Chen Y, Wang P, Zhang H (2021). PEO based polymer-ceramic hybrid solid electrolytes: a review. Nano Converg..

[68] Fan LZ, He HC, Nan CW (2021). Tailoring inorganic-polymer composites for the mass production of solid-state batteries. Nat. Rev. Mater..

[69] Zheng Y, Yao YZ, Ou JH, Li M, Luo D (2020). A review of composite solid-state electrolytes for lithium batteries: fundamentals, key materials and advanced structures. Chem. Soc. Rev..

[70] Tang S, Guo W, Fu YZ (2020). Advances in composite polymer electrolytes for lithium batteries and beyond. Adv. Energy Mater..

[71] Kalnaus S, Sabau AS, Tenhaeff WE, Dudney NJ, Daniel C (2012). Design of composite polymer electrolytes for Li ion batteries based on mechanical stability criteria. J. Power Sources.

[72] Zheng J, Wang PB, Liu HY, Hu YY (2019). Interface-enabled ion conduction in Li_10_GeP_2_S_12_-poly(ethylene oxide) hybrid electrolytes. ACS Appl. Energy Mater..

[73] Zheng J, Dang H, Feng XY, Chien PH, Hu YY (2017). Li-ion transport in a representative ceramic-polymer-plasticizer composite electrolyte: Li_7_La_3_Zr_2_O_12_–polyethylene oxide-tetraethylene glycol dimethyl ether. J. Mater. Chem. A.

[74] Liu SL, Liu WY, Ba DL, Zhao YZ, Ye YH (2023). Filler-integrated composite polymer electrolyte for solid-state lithium batteries. Adv. Mater..

[75] Chen L, Li YT, Li SP, Fan LZ, Nan CW (2018). PEO/garnet composite electrolytes for solid-state lithium batteries: from “ceramic-in-polymer” to “polymer-in-ceramic”. Nano Energy.

[76] Wang C, Yang TQ, Zhang WK, Huang H, Gan YP (2022). Hydrogen bonding enhanced SiO_2_/PEO composite electrolytes for solid-state lithium batteries. J. Mater. Chem. A.

[77] Zheng J, Hu YY (2018). New insights into the compositional dependence of Li-ion transport in polymer-ceramic composite electrolytes. ACS Appl. Mater. Interfaces.

[78] Wu N, Chien PH, Li YT, Dolocan A, Xu HH (2020). Fast Li^+^ conduction mechanism and interfacial chemistry of a NASICON/polymer composite electrolyte. J. Am. Chem. Soc..

[79] Chen H, Zheng M, Qian S, Ling HY, Wu Z (2021). Functional additives for solid polymer electrolytes in flexible and high-energy-density solid-state lithium-ion batteries. Carbon Energy.

[80] Su YX, Xu F, Qiu YQ, Zhang JB, Zhang XR (2022). Electrolyte based on laser-generated nano-garnet in poly(ethylene oxide) for solid-state lithium metal batteries. Chem. Eng. J..

[81] Pan QW, Zheng YW, Kota S, Huang WC, Wang SJ (2019). 2D MXene-containing polymer electrolytes for all-solid-state lithium metal batteries. Nanoscale Adv..

[82] Zhang XK, Xie J, Shi FF, Lin DC, Liu YY (2018). Vertically aligned and continuous nanoscale ceramic-polymer interfaces in composite solid polymer electrolytes for enhanced ionic conductivity. Nano Lett..

[83] Fan R, Liu C, He KQ, Cheng SH, Chen DZ (2020). Versatile strategy for realizing flexible room-temperature all-solid-state battery through a synergistic combination of salt affluent PEO and Li_6.75_La_3_Zr_1.75_Ta_0.25_O_12_ nanofibers. ACS Appl. Mater. Interfaces.

[84] Liu W, Lee SW, Lin DC, Shi FF, Wang S (2017). Enhancing ionic conductivity in composite polymer electrolytes with well-aligned ceramic nanowires. Nat. Energy.

[85] Sun ZJ, Li YH, Zhang SY, Shi L, Wu H (2019). g-C_3_N_4_ nanosheets enhanced solid polymer electrolytes with excellent electrochemical performance, mechanical properties, and thermal stability. J. Mater. Chem. A.

[86] An HW, Liu QS, An JL, Liang ST, Wang XF (2021). Coupling two-dimensional fillers with polymer chains in solid polymer electrolyte for room-temperature dendrite-free lithium-metal batteries. Energy Storage Mater..

[87] Shi YZ, Li B, Zhu Q, Shen K, Tang WJ (2020). MXene-based mesoporous nanosheets toward superior lithium ion conductors. Adv. Energy Mater..

[88] Tang WJ, Tang S, Zhang CJ, Ma QT, Xiang Q (2018). Simultaneously enhancing the thermal stability, mechanical modulus, and electrochemical performance of solid polymer electrolytes by incorporating 2D sheets. Adv. Energy Mater..

[89] Cheng J, Hou GM, Chen Q, Li DP, Li KK (2022). Sheet-like garnet structure design for upgrading PEO-based electrolyte. Chem. Eng. J..

[90] Li YH, Sun ZJ, Liu DY, Gao YY, Wang YK (2020). A composite solid polymer electrolyte incorporating MnO_2_ nanosheets with reinforced mechanical properties and electrochemical stability for lithium metal batteries. J. Mater. Chem. A.

[91] Li T, Ding B, Wang J, Qin ZY, Fernando JFS (2020). Sandwich-structured ordered mesoporous polydopamine/MXene hybrids as high-performance anodes for lithium-ion batteries. ACS Appl. Mater. Interfaces.

[92] Yu XW, Manthiram A (2020). A long cycle life, all-solid-state lithium battery with a ceramic-polymer composite electrolyte. ACS Appl. Energ. Mater..

[93] Cheng J, Hou GM, Sun Q, Liang Z, Xu XY (2020). Cold-pressing PEO/LAGP composite electrolyte for integrated all-solid-state lithium metal battery. Solid State Ion..

[94] Hou GM, Zhang MQ, Huang YF, Ruan WH (2016). A TiO_2_/PEO composite incorporated with in situ synthesized hyper-branched poly(amine-ester) and its application as a polymer electrolyte. RSC Adv..

[95] Isaac JA, Devaux D, Bouchet R (2022). Dense inorganic electrolyte particles as a lever to promote composite electrolyte conductivity. Nat. Mater..

[96] Zhang JX, Zhao N, Zhang M, Li YQ, Chu PK (2016). Flexible and ion-conducting membrane electrolytes for solid-state lithium batteries: Dispersion of garnet nanoparticles in insulating polyethylene oxide. Nano Energy.

[97] He KQ, Cheng SH, Hu JY, Zhang YQ, Yang HW (2021). In-situ intermolecular interaction in composite polymer electrolyte for ultralong life quasi-solid-state lithium metal batteries. Angew. Chem. Int. Ed..

[98] Li WW, Sun CZ, Jin J, Li YP, Chen CH (2019). Realization of the Li^+^ domain diffusion effect via constructing molecular brushes on the LLZTO surface and its application in all-solid-state lithium batteries. J. Mater. Chem. A.

[99] Wu N, Chien PH, Qian YM, Li YT, Xu HH (2020). Enhanced surface interactions enable fast Li^+^ conduction in oxide-polymer composite electrolyte. Angew. Chem. Int. Ed..

[100] Lin DC, Yuen PY, Liu YY, Liu W, Liu N (2018). A silica-aerogel-reinforced composite polymer electrolyte with high ionic conductivity and high modulus. Adv. Mater..

[101] Li Z, Sha WX, Guo X (2019). Three-dimensional garnet framework-reinforced solid composite electrolytes with high lithium-ion conductivity and excellent stability. ACS Appl. Mater. Interfaces.

[102] ZagóRski J, Amo JMLPD, Cordill MJ, Aguesse Fdr, Buannic L (2019). Garnet-polymer composite electrolytes: new insights on local Li-ion dynamics and electrodeposition stability with Li metal anodes. ACS Appl. Energy Mater..

[103] Liu M, Ganapathy S, Wagemaker M (2022). A direct view on Li-ion transport and Li-metal plating in inorganic and hybrid solid-state electrolytes. Acc. Chem. Res..

[104] Zheng J, Tang MX, Hu YY (2016). Lithium ion pathway within Li_7_La_3_Zr_2_O_12_–polyethylene oxide composite electrolytes. Angew. Chem. Int. Ed..

[105] Li MR, Kolek M, Frerichs JE, Sun W, Hou X (2021). Investigation of polymer/ceramic composite solid electrolyte system: the case of PEO/LGPS composite electrolytes. ACS Sustain. Chem. Eng..

[106] Zhu YZ, He XF, Mo YF (2016). First principles study on electrochemical and chemical stability of solid electrolyte-electrode interfaces in all-solid-state Li-ion batteries. J. Mater. Chem. A.

[107] Chi SS, Liu YC, Zhao N, Guo XX, Nan CW (2019). Solid polymer electrolyte soft interface layer with 3D lithium anode for all-solid-state lithium batteries. Energy Storage Mater..

[108] Pennycook SJ, Li CJ, Li MS, Tang CH, Okunishi E (2018). Material structure, properties, and dynamics through scanning transmission electron microscopy. J. Anal. Sci. Technol..

[109] Cheng Q, Li AJ, Li N, Li S, Zangiabadi A (2019). Stabilizing solid electrolyte-anode interface in Li-metal batteries by boron nitride-based nanocomposite coating. Joule.

[110] Liu M, Wang C, Cheng Z, Ganapathy S, Haverkate LA (2020). Controlling the lithium-metal growth to enable low-lithium-metal-excess all-solid-state lithium-metal batteries. ACS Mater. Lett..

[111] Cheng Y, Zhang LQ, Zhang QB, Li J, Tang YF (2020). Understanding all solid-state lithium batteries through in situ transmission electron microscopy. Mater. Today.

[112] Zhang CC, Feng YZ, Han Z, Gao S, Wang MY (2019). Electrochemical and structural analysis in all-solid-state lithium batteries by analytical electron microscopy: progress and perspectives. Adv. Mater..

[113] Chen H, Adekoya D, Hencz L, Ma J, Chen S (2020). Stable seamless interfaces and rapid ionic conductivity of Ca–CeO_2_/LiTFSI/PEO composite electrolyte for high-rate and high-voltage all-solid-state battery. Adv. Energy Mater..

[114] Xu HH, Chien PH, Shi JJ, Lia YT, Wu N (2019). High-performance all-solid-state batteries enabled by salt bonding to perovskite in poly(ethylene oxide). Proc. Natl. Acad. Sci. USA.

[115] Li X, Wang DH, Wang HC, Yan HF, Gong ZL (2019). Poly(ethylene oxide)–Li_10_SnP_2_S_12_ composite polymer electrolyte enables high-performance all-solid-state lithium sulfur battery. ACS Appl. Mater. Interfaces.

[116] Piana G, Bella F, Geobaldo F, Meligrana G, Gerbaldi C (2019). PEO/LAGP hybrid solid polymer electrolytes for ambient temperature lithium batteries by solvent-free, "one pot" preparation. J. Energy Storage.

[117] Wan ZP, Lei DN, Yang W, Liu C, Shi K (2019). Low resistance-integrated all-solid-state battery achieved by Li_7_La_3_Zr_2_O_12_ nanowire upgrading polyethylene oxide(PEO) composite electrolyte and PEO cathode binder. Adv. Funct. Mater..

[118] Li YT, Xu BY, Xu HH, Duan HN, Lv XJ (2017). Hybrid polymer/garnet electrolyte with a small interfacial resistance for lithium-ion batteries. Angew. Chem. Int. Ed..

[119] Li MR, Frerichs JE, Kolek M, Sun W, Zhou D (2020). Solid-state lithium-sulfur battery enabled by thio-LiSICON/polymer composite electrolyte and sulfurized polyacrylonitrile cathode. Adv. Funct. Mater..

[120] Huang ZY, Tong RA, Zhang J, Chen LH, Wang CA (2020). Blending poly(ethylene oxide) and Li_6.4_La_3_Zr_1.4_Ta_0.6_O_12_ by haake rheomixer without any solvent: a low-cost manufacture method for mass production of composite polymer electrolyte. J. Power Sources.

[121] Tong RA, Chen LH, Shao G, Wang HL, Wang CA (2021). An integrated solvent-free modification and composite process of Li_6.4_La_3_Zr_1.4_Ta_0.6_O12/poly(ethylene oxide) solid electrolytes: enhanced compatibility and cycle performance. J. Power Sources.

[122] Tong RA, Luo HL, Chen LH, Zhang JX, Shao G (2022). Constructing the lithium polymeric salt interfacial phase in composite solid-state electrolytes for enhancing cycle performance of lithium metal batteries. Chem. Eng. J..

[123] Huang ZY, Pang WY, Liang P, Jin ZH, Grundish N (2019). A dopamine modified Li_6.4_La_3_Zr_1.4_Ta_0.6_O_12_/PEO solid-state electrolyte: enhanced thermal and electrochemical properties. J. Mater. Chem. A.

[124] Chen L, Qiu XM, Bai ZM, Fan LZ (2021). Enhancing interfacial stability in solid-state lithium batteries with polymer/garnet solid electrolyte and composite cathode framework. J. Energy Chem..

[125] Ni'mah YL, Muhaiminah ZH, Suprapto S (2021). Increase of solid polymer electrolyte ionic conductivity using Nano-SiO_2_ synthesized from sugarcane bagasse as filler. Polymers.

[126] Wang X, Zhai HW, Qie BY, Cheng Q, Li AJ (2019). Rechargeable solid-state lithium metal batteries with vertically aligned ceramic nanoparticle/polymer composite electrolyte. Nano Energy.

[127] Pan P, Zhang MM, Cheng ZL, Jiang LY, Mao JT (2022). Garnet ceramic fabric-reinforced flexible composite solid electrolyte derived from silk template for safe and long-term stable all-solid-state lithium metal batteries. Energy Storage Mater..

[128] Li RG, Guo ST, Yu L, Wang LB, Wu DB (2019). Morphosynthesis of 3D macroporous garnet frameworks and perfusion of polymer-stabilized lithium salts for flexible solid-state hybrid electrolytes. Adv. Mater. Interfaces.

[129] Dai JQ, Fu K, Gong YH, Song JW, Chen CJ (2019). Flexible solid-state electrolyte with aligned nanostructures derived from wood. ACS Mater. Lett..

[130] Gong YH, Fu K, Xu SM, Dai JQ, Hamann TR (2018). Lithium-ion conductive ceramic textile: a new architecture for flexible solid-state lithium metal batteries. Mater. Today.

[131] Song SD, Qin XH, Ruan YL, Li WJ, Xu YQ (2020). Enhanced performance of solid-state lithium-air batteries with continuous 3D garnet network added composite polymer electrolyte. J. Power Sources.

[132] Fu XL, Li YC, Liao CZ, Gong WP, Yang MY (2019). Enhanced electrochemical performance of solid PEO/LiClO_4_ electrolytes with a 3D porous Li_6.28_La_3_Zr_2_Al_0.24_O_12_ network. Compos. Sci. Technol..

[133] Liu C, Wang JX, Kou WJ, Yang ZH, Zhai PF (2021). A flexible, ion-conducting solid electrolyte with vertically bicontinuous transfer channels toward high performance all-solid-state lithium batteries. Chem. Eng. J..

[134] Zekoll S, Marriner-Edwards C, Hekselman AKO, Kasemchainan J, Kuss C (2018). Hybrid electrolytes with 3D bicontinuous ordered ceramic and polymer microchannels for all-solid-state batteries. Energy Environ. Sci..

[135] Wang ZY, Shen L, Deng SG, Cui P, Yao XY (2021). 10 μm-thick high-strength solid polymer electrolytes with excellent interface compatibility for flexible all-solid-state lithium-metal batteries. Adv. Mater..

[136] Xie H, Yang CP, Fu K, Yao YG, Jiang F (2018). Flexible, scalable, and highly conductive garnet-polymer solid electrolyte templated by bacterial cellulose. Adv. Energy Mater..

[137] Zhai HW, Xu PY, Ning MQ, Cheng Q, Mandal J (2017). A flexible solid composite electrolyte with vertically aligned and connected ion-conducting nanoparticles for lithium batteries. Nano Lett..

[138] Li YH, Fu ZY, Lu SY, Sun X, Zhang XR (2022). Polymer nanofibers framework composite solid electrolyte with lithium dendrite suppression for long life all-solid-state lithium metal battery. Chem. Eng. J..

[139] Zhang MM, Pan P, Cheng ZL, Mao JT, Jiang LY (2021). Flexible, mechanically robust, solid-state electrolyte membrane with conducting oxide-enhanced 3D nanofiber networks for lithium batteries. Nano Lett..

[140] Zhang ZJ, Wang Q, Li ZH, Jiang YC, Zhao B (2019). Well-aligned BaTiO_3_ nanofibers via solution blow spinning and their application in lithium composite solid-state electrolyte. Mater. Express.

[141] He F, Tang WJ, Zhang XY, Deng LJ, Luo JY (2021). High energy density solid state lithium metal batteries enabled by sub-5 µm solid polymer electrolytes. Adv. Mater..

[142] Gao LX, Tang B, Jiang HY, Xie ZJ, Wei JP (2021). Fiber-reinforced composite polymer electrolytes for solid-state lithium batteries. Adv. Sustain. Syst..

[143] Navarra MA, Lombardo L, Bruni P, Morelli L, Tsurumaki A, Panero S (2018). Gel polymer electrolytes based on silica-added poly(ethylene oxide) electrospun membranes for lithium batteries. Membranes.

[144] Zha JW, Huang N, He KQ, Dang ZM, Shi CY (2017). Electrospun poly(ethylene oxide) nanofibrous composites with enhanced ionic conductivity as flexible solid polymer electrolytes. High Volt..

[145] Liu W, Liu N, Sun J, Hsu PC, Li YZ (2015). Ionic conductivity enhancement of polymer electrolytes with ceramic nanowire fillers. Nano Lett..

[146] Fu K, Gong YH, Dai JQ, Gong A, Han XG (2016). Flexible, solid-state, ion-conducting membrane with 3D garnet nanofiber networks for lithium batteries. Proc. Natl. Acad. Sci. USA.

[147] Wang XZ, Zhang YB, Zhang X, Liu T, Lin YH (2018). Lithium-salt-rich PEO/Li_0.3_La_0.557_TiO_3_ interpenetrating composite electrolyte with three-dimensional ceramic nano-backbone for all-solid-state lithium-ion batteries. ACS Appl. Mater. Interfaces.

[148] Yu JM, Wang C, Li SH, Liu N, Zhu J (2019). Li^+^-containing, continuous silica nanofibers for high Li^+^ conductivity in composite polymer electrolyte. Small.

[149] Wan JY, Xie J, Kong X, Liu Z, Liu K (2019). Ultrathin, flexible, solid polymer composite electrolyte enabled with aligned nanoporous host for lithium batteries. Nat. Nanotechnol..

[150] Cui Y, Wan JY, Ye YS, Liu K, Chou LY (2020). A fireproof, lightweight, polymer-polymer solid-state electrolyte for safe lithium batteries. Nano Lett..

[151] Wu JY, Rao ZX, Cheng ZX, Yuan LX, Li Z (2019). Ultrathin, flexible polymer electrolyte for cost-effective fabrication of all-solid-state lithium metal batteries. Adv. Energy Mater..

[152] Bae J, Li YT, Zhang J, Zhou XY, Zhao F (2018). A 3D nanostructured hydrogel-framework-derived high-performance composite polymer lithium-ion electrolyte. Angew. Chem. Int. Ed..

[153] Bae J, Li YT, Zhao F, Zhou XY, Ding Y (2018). Designing 3D nanostructured garnet frameworks for enhancing ionic conductivity and flexibility in composite polymer electrolytes for lithium batteries. Energy Storage Mater..

[154] Falco M, Castro L, Nair JR, Bella F, Bardé F (2019). UV-cross-linked composite polymer electrolyte for high-rate, ambient temperature lithium batteries. ACS Appl. Energy Mater..

[155] Siyal SH, Javed MS, Jatoi AH, Lan JL, Yu YH (2020). In situ curing technology for dual ceramic composed by organic-inorganic functional polymer gel electrolyte for dendritic-free and robust lithium-metal batteries. Adv. Mater. Interfaces.

[156] Siyal SH, Li MJ, Li H, Lan JL, Yu YH (2019). Ultraviolet irradiated PEO/LATP composite gel polymer electrolytes for lithium-metallic batteries (LMBs). Appl. Surf. Sci..

[157] Shi J, Xiong HG, Yang YF, Shao HX (2018). Nano-sized oxide filled composite PEO/PMMA/P(VDF-HFP) gel polymer electrolyte for rechargeable lithium and sodium batteries. Solid State Ion..

[158] Wang YT, Ju JW, Dong SM, Yan YY, Jiang F (2021). Facile design of sulfide-based all solid-state lithium metal battery: In situ polymerization within self-supported porous argyrodite skeleton. Adv. Funct. Mater..

[159] Wang RL, Dong Q, Wang CW, Hong M, Gao JL (2021). High-temperature ultrafast sintering: Exploiting a new kinetic region to fabricate porous solid-state electrolyte scaffolds. Adv. Mater..

[160] Zhang K, Wu F, Wang XR, Weng ST, Yang XY (2022). 8.5 µm-thick flexible-rigid hybrid solid-electrolyte/lithium integration for air-stable and interface-compatible all-solid-state lithium metal batteries. Adv. Energy Mater..

[161] Yu J, Lin XD, Liu JP, Yu JTT, Robson MJ (2021). In situ fabricated quasi-solid polymer electrolyte for high-energy-density lithium metal battery capable of subzero operation. Adv. Energy Mater..

[162] Chen Y, Huo F, Chen SM, Cai WB, Zhang SJ (2021). In-built quasi-solid-state poly-ether electrolytes enabling stable cycling of high-voltage and wide-temperature Li metal batteries. Adv. Funct. Mater..

[163] Chen DL, Zhu T, Zhu M, Yuan SQ, Kang PB (2022). In-situ constructing “ceramer” electrolytes with robust-flexible interfaces enabling long-cycling lithium metal batteries. Energy Storage Mater..

[164] Yang JX, Liu X, Wang Y, Zhou XW, Weng LT (2021). Electrolytes polymerization-induced cathode-electrolyte-interphase for high voltage lithium-ion batteries. Adv. Energy Mater..

[165] Shen H, Yi E, Heywood S, Parkinson DY, Chen GY (2020). Scalable freeze-tape-casting fabrication and pore structure analysis of 3D LLZO solid-state electrolytes. ACS Appl. Mater. Interfaces.

[166] Yi E, Shen H, Heywood S, Alvarado J, Parkinson DY (2020). All-solid-state batteries using rationally designed garnet electrolyte frameworks. ACS Appl. Energy Mater..

[167] Jiang TL, He PG, Wang GX, Shen Y, Nan CW (2020). Solvent-free synthesis of thin, flexible, nonflammable garnet-based composite solid electrolyte for all-solid-state lithium batteries. Adv. Energy Mater..

[168] Xu JJ (2022). Critical review on cathode-electrolyte interphase toward high-voltage cathodes for Li-ion batteries. Nanomicro Lett..

[169] Sheng OW, Jin CP, Luo JM, Yuan HD, Fang C (2017). Ionic conductivity promotion of polymer electrolyte with ionic liquid grafted oxides for all-solid-state lithium-sulfur batteries. J. Mater. Chem. A.

[170] Cao J, Wang L, He XM, Fang M, Gao J (2013). In situ prepared nano-crystalline TiO_2_–poly(methyl methacrylate) hybrid enhanced composite polymer electrolyte for Li-ion batteries. J. Mater. Chem. A.

[171] Huo HY, Zhao N, Sun JY, Du FM, Li YQ (2017). Composite electrolytes of polyethylene oxides/garnets interfacially wetted by ionic liquid for room-temperature solid-state lithium battery. J. Power Sources.

[172] Han FD, Westover AS, Yue J, Fan XL, Wang F (2019). High electronic conductivity as the origin of lithium dendrite formation within solid electrolytes. Nat. Energy.

[173] Kuhnert E, Ladenstein L, Jodlbauer A, Slugovc C, Trimmel G (2020). Lowering the interfacial resistance in Li_6.4_La_3_Zr_1.4_Ta_0.6_O_12_|poly(ethylene oxide) composite electrolytes. Cell Rep. Phys. Sci..

[174] Zhu YH, Cao J, Chen H, Yu QP, Li BH (2013). High electrochemical stability of 3D cross-linked network PEO@nano-SiO_2_ composite polymer electrolyte for lithium metal batteries. J. Mater. Chem. A.

[175] Kim Y, Kwon SJ, Jang HK, Jung BM, Lee SB (2017). High ion conducting nanohybrid solid polymer electrolytes via single-ion conducting mesoporous organosilica in poly(ethylene oxide). Chem. Mater..

[176] Choudhury S, Stalin S, Deng Y, Archer LA (2018). Soft colloidal glasses as solid-state electrolytes. Chem. Mater..

[177] Lin DC, Liu W, Liu YY, Lee HR, Hsu PC (2016). High ionic conductivity of composite solid polymer electrolyte via in situ synthesis of monodispersed SiO_2_ nanospheres in poly(ethylene oxide). Nano Lett..

[178] Tan XJ, Wu YM, Tang WP, Song SF, Yao JY (2020). Preparation of nanocomposite polymer electrolyte via in situ synthesis of SiO_2_ nanoparticles in PEO. Nanomaterials.

[179] Pan KC, Zhang L, Qian WW, Wu XK, Dong K (2020). A flexible ceramic/polymer hybrid solid electrolyte for solid-state lithium metal batteries. Adv. Mater..

[180] Bao WD, Zhao LQ, Zhao HJ, Su LX, Cai XC (2021). Vapor phase infiltration of ZnO quantum dots for all-solid-state PEO-based lithium batteries. Energy Storage Mater..

[181] Ma XN, Xu YL, Zhang BF, Xue X, Wang C (2020). Garnet Si-Li_7_La_3_Zr_2_O_12_ electrolyte with a durable, low resistance interface layer for all-solid-state lithium metal batteries. J. Power Sources.

[182] Chen LH, Zhang J, Tong RA, Zhang JX, Wang HL (2022). Excellent Li/garnet interface wettability achieved by porous hard carbon layer for solid state Li metal battery. Small.

[183] Liu K, Zhang RH, Sun J, Wu MC, Zhao TS (2019). Polyoxyethylene (PEO)|PEO−Perovskite|PEO composite electrolyte for all-solid-state lithium metal batteries. ACS Appl. Mater. Interfaces.

[184] Liang JN, Sun Q, Zhao Y, Sun YP, Wang CH (2018). Stabilization of all-solid-state Li-S batteries with a polymer-ceramic sandwich electrolyte by atomic layer deposition. J. Mater. Chem. A.

[185] Chen LH, Su YB, Zhang J, Zhang HJ, Fan BB (2021). Nanosecond laser cleaning method to reduce the surface inert layer and activate the garnet electrolyte for a solid-state Li metal battery. ACS Appl. Mater. Interfaces.

[186] Yang HC, Zhang YM, Tennenbaum MJ, Althouse Z, Ma Y (2019). Polypropylene carbonate-based adaptive buffer layer for stable interfaces of solid polymer lithium metal batteries. ACS Appl. Mater. Interfaces.

[187] Wang CH, Bai GL, Yang YF, Liu XJ, Shao HX (2019). Dendrite-free all-solid-state lithium batteries with lithium phosphorous oxynitride-modified lithium metal anode and composite solid electrolytes. Nano Res..

[188] Zhou WD, Wang ZX, Pu Y, Li YT, Xin S (2018). Double-layer polymer electrolyte for high-voltage all-solid-state rechargeable batteries. Adv. Mater..

[189] Zhang SZ, Liang TB, Wang DH, Xu YJ, Cui YL (2021). A stretchable and safe polymer electrolyte with a protecting-layer strategy for solid-state lithium metal batteries. Adv. Sci..

[190] Xu XY, Liu YY, Kapitanova OO, Song ZX, Sun J (2022). Electro-chemo-mechanical failure of solid electrolytes induced by growth of internal lithium filaments. Adv. Mater..

[191] Liu YY, Xu XY, Kapitanova OO, Evdokimov PV, Song ZX (2022). Electro-chemo-mechanical modeling of artificial solid electrolyte interphase to enable uniform electrodeposition of lithium metal anodes. Adv. Energy Mater..

[192] Liu L, Yang L, Liu M, Wang XY, Li XL (2019). A flexible tysonite-type La_0.95_Ba_0.05_F_2.95_@PEO-based composite electrolyte for the application of advanced fluoride ion battery. J. Energy Storage.

[193] Yang XF, Jiang M, Gao XJ, Bao DN, Sun Q (2020). Determining the limiting factor of the electrochemical stability window for PEO-based solid polymer electrolytes: main chain or terminal –OH group?. Energy Environ. Sci..

[194] Fan R, Liao WC, Fan SX, Chen DZ, Tang JN (2022). Regulating interfacial Li-ion transport via an integrated corrugated 3D skeleton in solid composite electrolyte for all-solid-state lithium metal batteries. Adv. Sci..

[195] Liu SJ, Shan HR, Xia SH, Yan JH, Yu JY (2020). Polymer template synthesis of flexible SiO_2_ nanofibers to upgrade composite electrolytes. ACS Appl. Mater. Interfaces.

[196] Sun JQ, He CH, Yao XM, Song AQ, Li YG (2020). Hierarchical composite-solid-electrolyte with high electrochemical stability and interfacial regulation for boosting ultra-stable lithium batteries. Adv. Funct. Mater..

[197] Wen X, Zeng QH, Guan JZ, Wen W, Chen PP (2022). 3D structural lithium alginate-based gel polymer electrolytes with superior high-rate long cycling performance for high-energy lithium metal batteries. J. Mater. Chem. A.

[198] Lopez J, Mackanic DG, Cui Y, Bao ZN (2019). Designing polymers for advanced battery chemistries. Nat. Rev. Mater..

[199] Judez X, Zhang H, Li CM, Eshetu GG, Zhang Y (2017). Polymer-rich composite electrolytes for all-solid-state Li-S cells. J. Phys. Chem. Lett..

[200] Fang RY, Xu HH, Xu BY, Li XY, Li YT (2020). Reaction mechanism optimization of solid-state Li–S batteries with a PEO-based electrolyte. Adv. Funct. Mater..

[201] Lee F, Tsai MC, Lin MH, Ni'mah YL, Hy S (2017). Capacity retention of lithium sulfur batteries enhanced with nano-sized TiO_2_-embedded polyethylene oxide. J. Mater. Chem. A.

[202] Tao XY, Liu YY, Liu W, Zhou GM, Zhao J (2017). Solid-state lithium–sulfur batteries operated at 37 °C with composites of nanostructured Li_7_La_3_Zr_2_O_12_/carbon foam and polymer. Nano Lett..

[203] Zhang YB, Chen RJ, Wang S, Liu T, Xu BQ (2020). Free-standing sulfide/polymer composite solid electrolyte membranes with high conductance for all-solid-state lithium batteries. Energy Storage Mater..

